# Elsinopirins A–D, Decalin Polyketides from the Ascomycete *Elsinoё pyri*

**DOI:** 10.3390/biom8010008

**Published:** 2018-02-05

**Authors:** Frank Surup, Kathrin Pommerehne, Hans-Josef Schroers, Marc Stadler

**Affiliations:** 1 Microbial Drugs, Helmholtz Centre for Infection Research GmbH (HZI), Inhoffenstraße 7, 38124 Braunschweig, Germany; frank.surup@helmholtz-hzi.de or frank.surup@gbf.de (F.S.); k.pommerehne@tu-braunschweig.de (K.P.); 2 German Centre for Infection Research Association (DZIF), Partner Site Hannover-Braunschweig, Inhoffenstraße 7, 38124 Braunschweig, Germany; 3 Institute of Biochemical Engineering, Technical University Braunschweig, Rebenring 56, 38106 Braunschweig, Germany; 4 Agricultural Institute of Slovenia, Hacquetova ulica 17, 1000 Ljubljana, Slovenia; hans.schroers@kis.si

**Keywords:** secondary metabolites, polyketides, structure elucidation, decalin

## Abstract

In course of our screening for new secondary metabolites from ecological niche specialized, phytopathogenic fungi, the plant pathogen *Elsinoё pyri*, strain 2203C, was found to produce four novel compounds (**1**–**4**), which were named elsinopirins A–D, in addition to the known metabolite elsinochrome A (**5**). After isolation by preparative high-performance liquid chromatography (HPLC), their structures, including relative stereochemistry, were elucidated by 1D and 2D nuclear magnetic resonance (NMR) and mass spectrometry (MS) data. Finally, absolute stereochemistry was assigned by chemical shifts of Mosher’s esters (α-methoxy-α-trifluoromethylphenylacetic acid; MTPA) derivatives of elsinopirin B (**2**). The compounds were found to be devoid of significant antibacterial, antifungal, and cytotoxic activities.

## 1. Introduction

**Figure 1:**
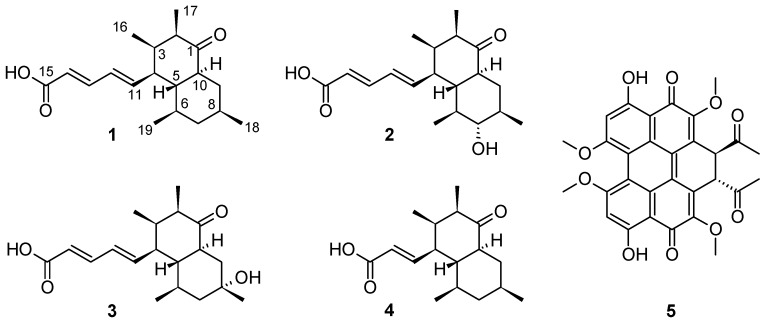
Structures of the secondary metabolites isolated from *Elsinoё piri* strain 2203C.

*Elsinoё pyri* is a fruit and leaf pathogen causing anthracnose disease symptoms and spots on apple and pear in temperate regions worldwide. The species is easily overlooked and its disease symptoms can be confused with the common pathogen *Venturia inaequalis*, the major apple scab agent in commercial production settings. Disease management strategies applied against *V. inaequalis* effectively suppress *E. pyri*, as the latter is commonly encountered in ecological production settings or private gardens [1,2,3]. The host specificity of Ep, its ability to compete with other plant cuticle-inhabiting fungi, and its slow in vitro growth triggered our interest in the secondary metabolite production of this fungus. According to our literature search, the species was hitherto untapped with respect to secondary metabolites. However, some pigments belonging to the class of perylene quinones named elsinochromes had been reported previously from a different species of *Elsinoё* [4,5]. We recently found moderate antimicrobial effects in cultures of strain 2202C, which was identified as *E. pyri* in our ongoing studies on bioactive compounds from plant-associated Ascomycota [6,7,8]. To assess its potential for secondary metabolite production, strain 2203C was cultivated on potato-dextrose-agar (Carl Roth GmbH + Co. KG, Karlsruhe, Germany) plate medium; extracts showed the presence of five metabolites that were isolated using preparative high-performance liquid chromatography (HPLC) (Figure 1). Herein we describe their isolation, structure elucidation, and biological characterization.

## 2. Results

### 2.1. Structure Elucidation

**Figure 2:**
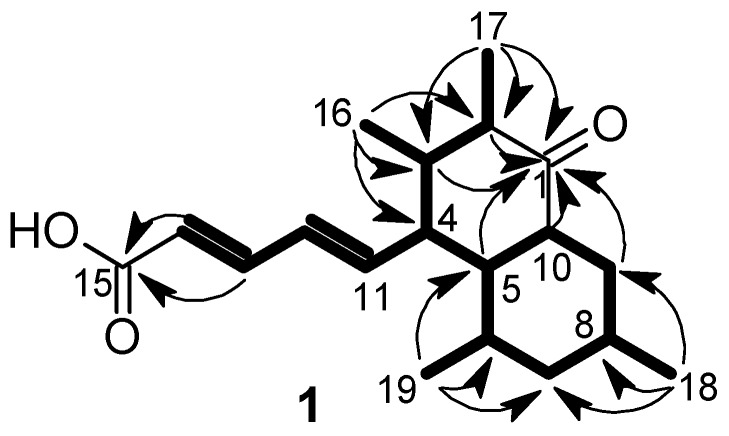
Selected correlation spectroscopy (COSY)/total correlated spectroscopy (TOCSY) (bold lines) and heteronuclear multiple bond (HMBC) (arrows) correlations indicative for the planar structure of elsinopirin A (**1**).

**Figure 3:**
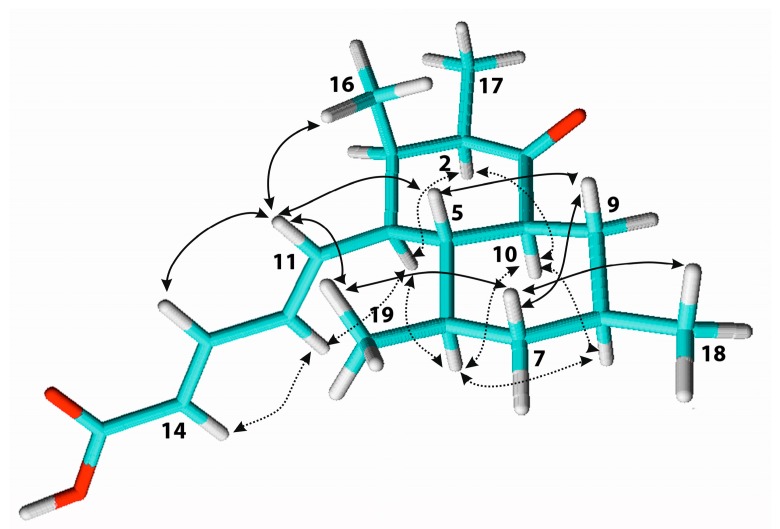
ROESY (rotating-frame nuclear Overhauser effect correlation spectroscopy) correlations indicative for the relative configuration of elsinopirin A (**1**); solid arrows are above main plane; dotted arrows are below.

Elsinopirin A (**1**) was isolated as a colorless oil; its HRESIMS (high-resolution electrospray ionization mass spectrometry) data indicated a molecular formula of C_19_H_28_O_3_, indicating a molecule with six degrees of unsaturation. ^1^H and HSQC (heteronuclear single quantum correlation spectroscopy) spectra revealed the presence of four methyl, two methylene, four olefinic, and seven aliphatic methine groups. The ^13^C spectrum furthermore indicated the presence of one ketone and one carboxylic acid. Starting from the methyls 16-H_3_, 17-H_3_, 18-H_3_, 19-H_3_ and the olefinic protons 11-H-15-H, one large spin system was assembled by ^1^H, ^1^H correlation spectroscopy (COSY) and total correlated spectroscopy (TOCSY) correlations (Figure 2). ^1^H,^13^C heteronuclear multiple bond (HMBC) correlations from 13-H/14-H to C-15 connected the carboxylic acid group to the trans diene (Δ^11,12^ 15.2 Hz, Δ^13,14^ 15.3 Hz), and correlations from 1-H, 2-H, 3-H, 17-H_3_ as well as 5-H, 9-H_2_, 10-H resulted in the assignment of the planar structure of **1**. The relative configuration was addressed by ^1^H,^1^H ROESY (rotating-frame nuclear Overhauser effect correlation spectroscopy) correlations and coupling constants. The large coupling constants of 4-H, 5-H,6-H, 7-Hb, 8-H, 9-Hb, 10-H around 10 Hz, which were confirmed by a *J*-resolved spectrum due to signal overlaps, indicated axial orientations of these atoms. Consequently, equatorial orientations of 18-H_3_, 19-H_3_ and the pentenoic side chain were deduced. ROESY correlations (Figure 3) were observed in ring B between 5-H, 7-Hb, 9-Hb on the β face of the molecule, and between 6-H, 8-H, and 10-H on the α face. For ring A, ROESY correlations between 2-H, 4-H, and 10-H indicated pseudoaxial orientations for these protons on the α face, resulting in equatorial orientations of 17-H_3_ and the pentenoic side chain. Moreover, a strong ROESY correlation was observed between 5-H and 16-H_3_ on the β face, indicating an axial orientation of 16-H_3_. Since the absolute stereochemistry of the metabolite family was established with **2** by Mosher’s method (see below), the configuration of elsinopirin A (**1**) was assigned as 2*R*, 3*R*, 4*S*, 5*R*, 6*R*, 8*S*, 10*S*.

**Figure 4:**
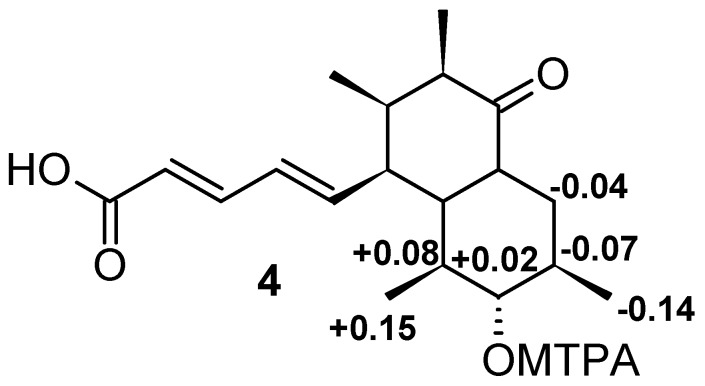
Δδ^SR^ chemical shifts of MTPA (α-methoxy-α-trifluoromethylphenylacetic acid) (Mosher) derivatives of elsinopirin B (**2**).

The molecular formula C_19_H_28_O_4_ of elsinopirin B (**2**) indicated the presence of an additional oxygen atom compared to **1**. The nuclear magnetic resonance (NMR) spectra of **2** were highly similar to those of **1**, with the key difference being the replacement of one methylene by an oxymethine group. Since both methyls 18-H_3_ and 19-H_3_ showed HMBC correlations to this additional oxymethine, it was identified as C-7. Because 7-H shows two large coupling constants around 9 Hz to both 6-H and 8-H, and ROESY correlations to 5-H/18-H_3_/19-H_3_, a β-axial orientation was assigned for 7-H. The secondary hydroxyl function of **2** was utilized to assign the absolute configuration of the elsinopyirin family by Mosher’s method. The positive Δδ^SR^ values of 6-H/19-H_3_ and negative ones for 8-H/9-H_2_/18-H_3_ were the characteristic for a 7*R* configuration (Figure 4). In this experiment, COSY and TOCSY determined the chemical shifts of protons in the vicinity of the secondary alcohol function of C-7. An HMBC correlation from 18-H_3_ to methylene C-9, which was clearly identified by a phase-sensitive HSQC experiment, was utilized to distinguish 18-H_3_ from 19-H_3_.

Elsinopirin C (**3**) was analyzed for the same molecular formula C_19_H_28_O_4_ as elsinopirin B (**2**). Again, the NMR spectra of **3** were highly similar to those of **1**. The key difference was identified in the replacement of a methine by an oxygenated quaternary carbon atom. This was located to be C-8 by an HMBC correlation of 18-H_3_. Because 8-OH exhibits ROESY correlations to 6-H and 10-H, whereas 18-H_3_ correlated to 7-H_a_ and 9-H_a_, an 8*R* configuration was assigned for C-8.

Elsinopirin D (**4**) was analyzed as C_17_H_26_O_3_ by HRESIMS. This accounted for the formal loss of a C_2_H_2_ fragment compared to the parental metabolite **1**. The NMR spectra of **4** were highly similar to those of **1**, with the key difference being the presence of only two olefinic methines instead of four. Consequently, **4** was assigned the same metabolite core as **1**, but bearing a propenoic acid instead of the pentadienoic side chain. Nearly identical ^13^C chemical shifts indicated identical relative and absolute configurations.

### 2.2. Semisynthetic Conversions

**Figure 5:**
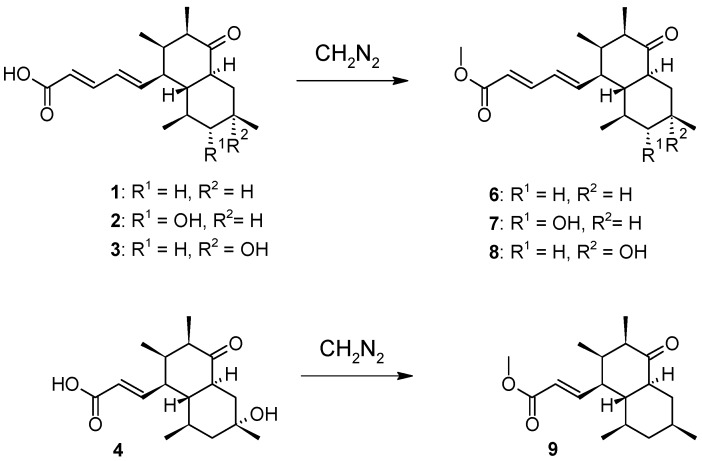
Semisynthetic conversion of elsinopirins A–D (**1**–**4**) into their methyl esters **6**–**9**.

Since a methyl ester moiety was proven to be necessary for the activity of a structurally close relative of **1**–**4** [9], methyl esters of elsinopirins A–D (**6**–**9**) were generated by the methylation of **1**–**4** through diazomethane (Figure 5).

### 2.3. Biological Activity

The biological activity of metabolites **1**–**5** was assessed against a broad test panel of bacteria and fungi as well as the cell lines L929 and KB3.1 [10]. However, no inhibition was observed for the Gram-positive bacteria *Bacillus subtilis*, *Micrococcus luteus*, *Mycobacterium* sp., *Staphylococcus aureus*, the Gram-negative bacteria *Chromobacterium violaceum*, *Escherichia coli*, *Pseudomonas aeruginosa*, the filamentous fungus *Mucor hiemalis*, and the yeasts *Candida albicans*, *Pichia anomala*, *Rhodotorula glutinis*, and *Schizosaccharomyces pombe* up to 66 µg/mL. In the same assay, **5** was active against *Mucor hiemalis* (Minimum inhibitory concentrations (MIC) = 16.6 µg/mL), *B. subtilis* (MIC = 16.6 µg/mL), and *S. aureus* (MIC = 8.3 µg/mL). Compounds **1a**–**4a** were tested only against *S. aureus* due to low substance amounts, but were inactive up to 100 µm/mL.

Only a very weak activity was observed for **1** against the cell line KB3.1 (inhibitory concentration of 50% (IC_50_) = 37 µg/mL), whereas no activity at all was observed for compounds **2**–**4** and **1a**–**4a** up to 66 µg/mL. In contrast, elsinochrome A (**5**) showed cytotoxic effects of IC_50_ = 4 µg/mL against L929 and 1.1 µg/mL against KB3.1 cells.

## 3. Discussion

Elsinopirins A–D (**1**–**4**) belong to a group of metabolites containing the ‘decalin’ motif [11]. This group comprises compounds from the isoprenoid and polyketide pathways and includes the famous compound lovastatin. Interestingly, the polyketide decalin skeleton is believed to be biosynthesized by an enzymatic intramolecular Diels-Alder reaction. More specifically, **1**–**4** belong to a subgroup possessing a penta-2,4-dienoic side chain connected to the decalin core. The majority of these were detected in cultures of *Penicillium* species and show a plethora of interesting bioactivities. Tanzawaic acid B from *Penicillium citrinum* inhibited superoxide anion production in human neutrophils [12]. Tanzawaic acids A, E, and K showed inhibition of the conidial germination in the rice blast fungus *Magnaporthe oryzae* in concentrations of about 25 µg/mL [13]. Hynapenes A–C, isolated from a soil-inhabiting *Penicillium* species, were effective against monensin-resisant *Eimeria tenella* [14].

However, no bioactivity could be detected for the elsinopirins A–D (**1**–**4**). In the case of the structurally closely related metabolite coprophilin, the metabolites carboxylic acid moiety was methylated and showed anticoccidial activity [9], whereas its free acid derivative was inactive. It was speculated that the differences in biological activity are mainly based on differences in cellular uptake. Consequently, the free carboxylic acids **1**–**4** were methylated by diazomethane. However, the methyl ester derivatives **6**–**9** did not show any activity in our broad test panel either. Therefore, we cannot speculate deeply about the biological function of these metabolites.

## 4. Materials and Methods

### 4.1. General

Optical rotations were determined with a Perkin-Elmer (Waitham, Middlesex County, MA, USA) 241 spectrometer and ultraviolet (UV) spectra were recorded with a Shimadzu (Shimadzu, Kyoto, Japan) UV-Vis spectrophotometer UV-2450. NMR spectra were recorded with Bruker Avance (Bruker, Bremen, Germany) III 700 spectrometer with a 5 mm TCI CryoProbe Bruker (^1^H 700 MHz, ^13^C 175 MHz) and Bruker Avance III 500 (^1^H 500 MHz, ^13^C 125 MHz) spectrometers. Chemical shifts δ were referenced to the solvents: chloroform-*d* (^1^H, δ = 7.27 ppm; ^13^C, δ = 77.0 ppm), pyridine-*d*_5_ (^1^H, δ = 7.22 ppm; ^13^C, δ = 123.9 ppm), DMSO-*d*_6_ (^1^H, δ = 2.50 ppm; ^13^C, δ = 49.15 ppm). Standard pulse programs provided by Bruker were utilized: ^1^H: zg30, ^13^C: zgpg30, COSY: cosyfpppqf, Jresolved: jresqf, TOCSY: mlevphpp, ROESY: roesyphpp.2 (mixing time: 500 ms), HSQC: hsqcedetgpsisp2.2, HMBC: hmbcgplpndqf. ESIMS and HRESIMS mass spectra were obtained with an Agilent (Waldbronn, Germany) 1200 series HPLC-UV system (column 2.1 × 50 mm, 1.7 µm, C18 Acquity UPLC BEH (Waters, Milford, MA, USA), solvent A: H_2_O + 0.1% formic acid; solvent B: AcCN + 0.1% formic acid, gradient: 5% B for 0.5 min increasing to 100% B in 19.5 min, maintaining 100% B for 5 min, flow rate = 0.6 mL/min, UV-visible detection 200–600 nm) combined with electrospray ion trap mass spectrometry (amaZon SL ion trap, Bruker) and electrospray ionisation time-of-flight mass spectrometry (ESI-TOF-MS) (maXis II, Bruker), respectively. Isolation of pure compounds was achieved with a model 2020 preparative HPLC system (Gilson, Middleton, WI, USA). A VP Nucleodur C18 ec column (250 × 21 mm, 5 µm, Macherey-Nagel, Düren, Germany) with 15 mL/min was used as the stationary phase. The mobile phase was composed of deionized water (Milli-Q, Millipore, Schwalbach, Germany; solvent A) and acetonitrile (AcCN, solvent B).

### 4.2. Fungal Material

Ripe apples showing the colonization of sooty blotch fungi and scab-like symptoms were collected in Vesela Gora, Slovenia, by H. J. Schroers in October 2009. The *E. pyri* strain 2203C was isolated by Ajda Medjedović. Taxonomic identification was based on morphological characters and confirmed by generating the DNA barcode sequence of the internal transcribed spacer regions 1 and 2 and the 5.8S rDNA of the rRNA gene cluster using reference sequences KX887267 and KX887268 from Gen Bank (www.ncbi.nlm.nih.gov) of strains CBS 163.29 and CBS 179.82 [3].

### 4.3. Fermentation and Downstream Processing

The solid-state cultivation of strain 2203C was carried out on PD agar plates (Potato Dextrose Agar, Carl Roth). The plates (ca. 30 mL medium) were inoculated with 3 mL of a liquid pre-culture in YMG medium (1.0% malt extract, 0.4% glucose, 0.4% yeast extract, pH 6.3). The agar of the culture changed to a red color and was harvested after 14 days of growth at 23 °C.

The agar plates were cut into small pieces and poured into a glass bottle, overlaid with ca. 100 mL of ethyl acetate. The bottle was then incubated in an ultrasonic bath for 30 min. Thereafter, the ethyl acetate was filtered and evaporated *in vacuo* to yield 72.7 mg of an oily crude extract.

The crude extract was dissolved in 1 mL of methanol and filtered through a reversed phase solid phase cartridge (Strata-X 33 mm, Polymeric Reversed Phase; Phenomenex, Aschaffenburg, Germany). The filtrate was subsequently subjected to preparative RP HPLC using a Kromasil precolumn (Kromasil, Amsterdam, The Netherlands, 100 C18; 50 × 20 mm; 7 µm) and a Nucleodur column (Nucleodur 100 C18ec; 250 × 21 mm; 5 µm) with the following conditions: mobile phase with solvent A (deionized water) and solvent B (acetonitrile), linear gradient of solvent B from 40 to 80% in 45 min, an increase to 100% in 5 min, followed by isocratic conditions at 100% solvent B for 6 min, flow rate of 15 mL/min, UV peak detection at 254 and 210 nm. The obtained fractions were combined according to the main peaks, leading to four main fractions which were evaporated and analyzed by HPLC-MS. The fraction with a retention time of 22 min contained **4** (1.6 mg), the one with 28 min contained **1** (1.8 mg), and the one with 34 min contained metabolite **5** (2.0 mg). The first fraction, which was eluted at around 10 min, contained two different substances (**2** and **3**) and was therefore forwarded to another preparative HPLC. The conditions were the same as before, besides the gradient which was set to a linear gradient of solvent B from 20 to 45% followed by an increase to 100% in 5 min and isocratic conditions for 6 min. The fractions were again combined according to the peaks, evaporated, and analyzed by HPLC-MS to yield **2** (2.4 mg) and **3** (2.7 mg). Five pure compounds were finally obtained, and their physicochemical characteristics are summarized below. NMR and mass spectra are provided in the Supplementary Materials.

#### 4.3.1. Elsinopirin A (**1**)

Colorless oil. [α]^25^_D_ = +3.1 (c 0.2, MeOH). UV (MeOH) λ_max_ (log ε): 262 nm (4.38). ^13^C NMR (175 MHz, CDCl_3_): see Table 1; ^1^H NMR (700 MHz, CDCl_3_): see Table 2. HRESIMS *m*/*z* 305.2110 ([M + H]^+^, calcd for C_19_H_29_O_3_ 305.2111).

#### 4.3.2. Elsinopirin B (**2**)

Colorless oil. [α]^25^_D_ = +9.7 (c 0.2, MeOH). UV (MeOH) λ_max_ (log ε): 262 nm (4.43). ^1^H NMR (700 MHz, DMSO-*d*_6_): see Table 2; ^13^C NMR (175 MHz, DMSO-*d*_6_): see Table 1; ^1^H NMR (pyridine-*d*_5_, 700 MHz): δ_H_ 7.76 (dd, *J* = 15.0 Hz, 10.5 Hz, 13-H), 6.29–6.38 (m, 11-H, 12-H, 14-H), 2.74–2.82 (m, 2-H, 7-H), 2.73 (td, *J* = 9.7 Hz, 3.0 Hz, 4-H), 2.16 (ddd, *J* = 12.0 Hz, 11.0 Hz, 2.5 Hz, 10-H), 2.07 (dt, *J* = 13.9 Hz, 3.3 Hz, 9-H_a_), 1.98 (m, H-3), 1.62 (m, 6-H), 1.57 (m, 8-H), 1.54 (m, 5-H), 1.33 (d, *J* = 6.2 Hz, 19-H), 1.23 (d, *J* = 6.5 Hz, 18-H), 1.29 (dt, *J* = 13.9 Hz, 12.0 Hz, 9-H_b_), 1.02 (d, *J* = 6.7 Hz, 17-H), 0.68 (d, *J* = 7.3 Hz, 16-H); ^13^C NMR (pyridine-*d*_5_, 175 MHz): δ_C_ 211.5 (C-1), 148.8* (C-11), 144.2 (C-13), 127.4 (C-12), 122.3 * (C-14), 79.9 (C-7), 53.3 (C-10), 51.6 (C-4), 48.5 (C-2), 48.2 (C-5), 46.8 (C-6), 45.7 (C-3), 38.9 (C-8), 32.6 (C-9), 19.7 (C-18), 18.1 (C-19), 11.7 (C-17), 8.8 (C-16), *: these chemical shifts were extracted from a ^1^H,^13^C HSQC spectrum. HRESIMS *m*/*z* 321.2059 ([M + H] ^+^, calcd for C_19_H_29_O_4_ 321.2060).

#### 4.3.3. Elsinopirin C (**3**)

Colorless oil. [α]^25^_D_ = +12.7 (c 0.3, MeOH). UV (MeOH) λ_max_ (log ε): 262 nm (4.40). ^1^H NMR (700 MHz, DMSO-*d*_6_): see Table 2; ^13^C NMR (175 MHz, DMSO-*d*_6_): see Table 1. HRESIMS *m*/*z* 321.2061 ([M + H]^+^, calcd for C_19_H_29_O_3_ 305.2060).

#### 4.3.4. Elsinopirin D (**4**)

Colorless oil. [α]^25^_D_ =+ 2.7 (c 0.1, MeOH). UV (MeOH) λ_max_ (log ε): 212 nm (4.23). ^1^H NMR (700 MHz, CDCl_3_): see Table 2; ^13^C NMR (175 MHz, CDCl_3_): see Table 1. HRESIMS *m*/*z* 279.1953 ([M + H]^+^, calcd for C_17_H_27_O_3_ 279.1955).

#### 4.3.5. Elsinochrome A (**5**)

Bright red oil. UV (H_2_O/ACN + 0.1% trifluoric acid (TFA)) λ_max_: 224, 268, 338, 456, 528, 566 nm. ^1^H NMR (500 MHz, DMSO-*d*_6_): δ_H_ 16.30 (s, 2H, 4-OH, 11-OH), 6.94 (s, 2H, 6-H, 9-H), 5.19 (s, 2H, 1-H, 2-H), 4.19 (s, 6H, 17-H_3_, 20-H_3_), 4.13 (s, 6H, 18-H_3_, 19-H_3_), 2.13 (s, 6H, 14-H_3_, 16-H_3_); ^13^C NMR (125 MHz, DMSO-*d*_6_): δ_C_ 204.3, 177.4, 173.3, 167.4, 149.7, 130.1, 122.0, 121.9, 117.3, 106.5, 102.2, 60.8, 57.3, 48.0, 27.9. ESIMS *m*/*z* 545.17 [M + H]^+^, 543.11 [M − H]^−^.

### 4.4. Semisynthetic Conversions

Diazomethane (0.4 mmol) in ether (1 mL), which was freshly produced in a Sigma-Aldrich (Sigma-Aldrich Corporation, Deisenhofen, Germany) Diazomethane Generator according to the manufacturer’s instructions, was added stepwise to **1** (1.0 mg) in aqueous methanol (50%, 1 mL) at 0 °C. The reaction was monitored by analytical HPLC and stopped by removing the solvent *in vacuo* after all starting material had been converted. This provided the methyl ester of elsinopirin A (**6**) without further purification.

The methyl esters **7**–**9** of elsinopirins B–D (**2**–**4**) were obtained analogously.

#### 4.4.1. Elsinopirin A Methyl Ester (**6**)

Colorless oil. UV (H_2_O/ACN + 0.1% TFA) λ_max_: 270 nm. ^1^H NMR (700 MHz, DMSO-*d*_6_): δ_H_ 7.29 (dd, *J* = 15.3, 11.0 Hz, 13-H), 6.48 (dd, *J* = 15.3, 9.7 Hz, 11-H), 6.31 (dd, *J* = 15.3, 11.0 Hz, 12-H), 5.91 (d, *J* = 15.3 Hz, 14-H), 3.65 (s, O*C*H_3_), 2.88 (qd, *J* = 6.7, 5.5 Hz, 2-H), 2.78 (ddd, *J* = 10.5, 9.7, 3.9 Hz, 4-H), 2.20 (br dd, *J* = 12.0, 11.0 Hz, 10-H), 1.99 (m, 3-H), 1.77 (m, 9-H_a_), 1.53 (m, 7-H_a_), 1.46 (m, 6-H), 1.37 (m, 8-H), 1.31 (ddd, *J* = 11.0, 10.5, 9.5 Hz, 5-H), 0.87 (d, *J* = 6.5 Hz, 19-H_3_) *, 0.85 (d, *J* = 6.5 Hz, 18-H_3_) *, 0.83 (d, *J* = 6.7 Hz, 17-H_3_), 0.79 (dt, *J* = 13.8, 12.0 Hz, 9-H_b_), 0.67 (dt, *J* = 13.7, 11.5 Hz, 7-H_b_), 0.60 (d, *J* = 7.1 Hz, 16-H_3_) * these assignments could be interchanged. ESIMS *m*/*z* 341.22 [M + Na]^+^, 319.24 [M + H]^+^.

#### 4.4.2. Elsinopirin B Methyl Ester (**7**)

Colorless oil. UV (H_2_O/ACN + 0.1% TFA) λ_max_: 271 nm. ^1^H NMR (700 MHz, DMSO-*d*_6_): δ_H_ 7.29 (dd, *J* = 15.3, 10.8 Hz, 13-H), 6.49 (dd, *J* = 15.3, 9.7 Hz, 11-H), 6.30 (dd, *J* = 15.3, 10.8 Hz, 12-H), 5.91 (d, *J* = 15.3 Hz, 14-H), 4.44 (br d, *J* = 6.7 Hz, 7-OH), 3.65 (s, O*C*H_3_), 2.88 (qd, *J* = 6.8, 5.5 Hz, 2-H), 2.81 (ddd, *J* = 10.5, 9.7, 3.6 Hz, 4-H), 2.38 (m, 7-H), 2.23 (br dd, *J* = 12.0, 11.0 Hz, 10-H), 1.99 (m, 3-H), 1.75 (dt, *J* = 13.8, 3.2 Hz, 9-H_a_), 1.46 (ddd, *J* = 11.0, 10.5, 9.5 Hz, 5-H), 1.29 (m, 6-H), 1.24 (m, 8-H), 0.96 (d, *J* = 6.5 Hz, 19-H_3_), 0.94 (d, *J* = 6.5 Hz, 18-H_3_), 0.90 (m, 9-H_b_), 0.83 (d, *J* = 6.8 Hz, 17-H_3_), 0.59 (d, *J* = 7.1 Hz, 16-H_3_). ESIMS *m*/*z* 357.22 [M + Na]^+^, 335.23 [M + H]^+^, 317.22 [M + H − H_2_O]^+^.

#### 4.4.3. Elsinopirin C Methyl Ester (**8**)

Colorless oil. UV (H_2_O/ACN + 0.1% TFA) λ_max_: 271 nm. ^1^H NMR (700 MHz, DMSO-*d*_6_): δ_H_ 7.28 (dd, *J* = 15.3, 10.8 Hz, 13-H), 6.48 (dd, *J* = 15.3, 9.6 Hz, 11-H), 6.32 (dd, *J* = 15.3, 10.8 Hz, 12-H), 5.91 (d, *J* = 15.3 Hz, 14-H), 3.65 (s, O*C*H_3_), 2.91 (qd, *J* = 6.5, 5.5 Hz, 2-H), 2.82 (ddd, *J* = 10.5, 9.6, 3.7 Hz, 4-H), 2.54 (m, 10-H), 1.98 (m, 3-H), 1.80 (m, 6-H), 1.68 (dt, *J* = 13.8, 2.5, 9-H_a_), 1.43 (dt, *J* = 13.7, 3.2 Hz, 7-H_a_), 1.31 (ddd, *J* = 11.0, 10.5, 9.8 Hz, 5-H), 1.15 (dd, *J* = 13.8, 12.2 Hz, 9-H_b_), 1.09 (s, 18-H_3_), 1.43 (dd, *J* = 13.7, 12.2 Hz, 7-H_b_), 0.83 (m, 19-H_3_) *, 0.82 (m, 17-H_3_) *, 0.83 (d, *J* = 6.8 Hz, 18-H_3_), 0.59 (d, *J* = 7.1 Hz, 16-H_3_), * these assignments could be interchanged. ESIMS *m*/*z* 357.21 [M + Na]^+^, 317.22 [M + H − H_2_O]^+^.

#### 4.4.4. Elsinopirin D Methyl Ester (**9**)

Colorless oil. UV (H_2_O/ACN + 0.1% TFA) λ_max_: 225 nm. ^1^H NMR (700 MHz, DMSO-*d*_6_): δ_H_ 7.05 (dd, *J* = 15.7, 9.8 Hz, 11-H), 5.95 (d, *J* = 15.7 Hz, 12-H), 3.65 (s, O*C*H_3_), 2.86–2.92 (m, 2-H, 4-H), 2.20 (br dd, *J* = 12.0, 11.0 Hz, 10-H), 2.02 (qdd, *J* = 7.1, 5.5, 4.0 Hz, 3-H), 1.76 (m, 9-H_a_), 1.53 (m, 7-H_a_), 1.46 (m, 6-H), 1.39 (ddd, *J* = 11.0, 10.5, 9.5 Hz, 5-H), 1.37 (m, 8-H), 0.85 (d, *J* = 6.5 Hz, 19-H_3_) *, 0.84 (d, *J* = 6.5 Hz, 18-H_3_) *, 0.83 (d, *J* = 6.7 Hz, 17-H_3_) *, 0.80 (dt, *J* = 13.8, 12.0 Hz, 9-H_b_), 0.67 (dt, *J* = 13.4, 12.0 Hz, 7-H_b_), 0.58 (d, *J* = 7.1 Hz, 16-H_3_) * these assignments could be interchanged. ESIMS *m*/*z* 315.20 [M + Na]^+^, 261.17 [M + H − H_2_O]^+^.

### 4.5. Mosher’s Analyses

For the preparation of the (*S*)-MTPA ester, 0.8 mg of **2** was dissolved in 600 µL of pyridine-*d*_5_, and 10 µL of (*R*)-MTPA chloride was added. The mixture was incubated at 25 °C for 10 min before the measurement of ^1^H, COSY, TOCSY, HSQC and HMBC NMR spectra: ^1^H NMR (700 MHz, pyridine-*d*_5_): similar to **2**, but δ_H_ 4.71 (7-H), 2.00 (9-H_a_), 1.73 (6-H), 1.57 (8-H), 0.98 (19-H_3_), 0.82 (18-H_3_). The (*R*)-MTPA ester was prepared in the same manner by the addition of 10 µL of (*S*)-MTPA chloride: ^1^H NMR (700 MHz, pyridine-*d*_5_): similar to **2**, but δ_H_ 4.69 (7-H), 2.04 (9-H_a_), 1.65 (6-H), 1.64 (8-H), 0.96 (18-H_3_), 0.83 (19-H_3_).

### 4.6. Serial Dilution and Cytotoxicity Assay

Minimum inhibitory concentrations were determined in a serial dilution assay carried out in a manner similar to that previously described [10]. Various test organisms were used to assess the antibacterial and antifungal activities. The *in vitro* cytotoxicity assay with the mouse cell line L929 was performed as previously described [10].

## 5. Conclusions

In summary, we isolated four novel metabolites from *E. pyri* strain 2203C, and elucidated their structures including absolute stereochemistry. Since no significant bioactivity was detected, their ecological role remains elusive.

## Figures and Tables

**Table 1:** ^13^C chemical shifts (175 MHz for **1**, **2**, **4**; 125 MHz for **3**) of elsinopirins A–D (**1**–**4**).

	1 ^a^	2 ^b^	3 ^b^	4 ^a^
1	212.5, qC	211.8, qC	212.6, qC	212.1, qC
2	49.0, CH	47.5, CH	47.8, CH	48.9, CH
3	45.9, CH	45.1, CH	45.4, CH	45.9, CH
4	51.8, CH	50.6, CH	50.2, CH	50.9, CH
5	49.4, CH	47.5, CH	48.5, CH	49.2, CH
6	39.1, CH	45.6, CH	33.9, CH	38.9, CH
7	45.9, CH_2_	79.1, CH	49.0, CH_2_	45.7, CH_2_
8	31.3, CH	37.9, CH	67.0, qC	31.3, CH
9	33.8, CH_2_	32.0, CH_2_	38.0, CH_2_	33.8, CH_2_
10	54.2, CH	52.2, CH	48.6, CH	54.1, CH
11	151.0, CH	149.8, CH	149.9, CH	157.4, CH
12	126.8, CH	126.5, CH	126.7, CH	118.9, CH
13	147.0, CH	144.5, CH	144.4, CH	169.9, qC
14	118.5, CH	120.4, CH	120.6, CH	
15	170.8, qC	167.8. qC	167.8, qC	
16	8.9, CH_3_	8.7, CH_3_	8.6, CH_3_	8.9, CH_3_
17	11.6, CH_3_	11.4, CH_3_	11.6, CH_3_	11.5, CH_3_
18	22.4, CH_3_	19.5, CH_3_	31.3, CH_3_	22.3, CH_3_
19	22.7. CH_3_	17.6, CH_3_	22.0, CH_3_	22.4, CH_3_

^a^ in CDCl_3_, ^b^ in DMSO-*d*_6_.

**Table 2:** ^1^H chemical shifts (700 MHz) of elsinopirins A–D (**1**–**4**).

	1 ^a^	2 ^b^	3 ^b^	4 ^a^
2	2.79, qd (6.7, 5.5)	2.88, qd (6.7, 5.5)	2.91, dq (8.5, 6.5)	2.80, m
3	2.09, m	1.99, m	1.98, dqd (8.5, 6.5, 3.4)	2.11, m
4	2.72, ddd (10.5, 9.5, 3.6)	2.80, ddd (10.5, 9.3, 3.4)	2.81, ddd (10.5, 9.3, 3.4)	2.78, m
5	1.39, ddd (10.5,10.0, 9.0)	1.30, ddd (11.0, 10.5, 9.8)	1.30, ddd (11.0, 10.5, 9.8)	1.44, ddd (10.5,10.0, 9.0)
6	1.49, m	1.30, m	1.81, m	1.49, m
7	1.61, br d (14.0)	2.39, m	1.43, br d (13.3)	1.62, br d (13.4)
	0.73, dt (14.0, 12.0)	OH: 4.44, br d (6.0)	1.00, dd (13.3, 12.2)	0.75, m
8	1.41, m	1.23, m		1.41, m
9	1.93, br d (13.9)	1.75, br d (13.4)	1.67, br d (13.4)	1.94, br d (13.6)
	0.92, m	0.90, dt (13.4, 12.0)	1.15, dd (13.4, 12.4)	0.95, m
10	2.08, m	2.23, dd (12.0, 11.0)	2.52, m	2.09, m
11	6.33, dd (15.2, 9.5)	6.41, dd (15.4, 9.3)	6.39, dd (15.0, 9.3)	7.22, dd (15.5, 10.0)
12	6.24, dd (15.2, 11.0)	6.27, dd (15.4, 10.8)	6.29, dd (15.0, 10.8)	5.88, d (15.5)
13	7.37, dd (15.3, 11.0)	7.19, dd (15.3, 10.8)	7.18, dd (15.3, 10.8)	
14	5.84, d (15.3)	5.80, d (15.3)	5.80, d (15.3)	
16	0.70, d (7.1)	0.59, d (7.1)	0.59, d (7.2)	0.73, d (7.1)
17	0.98, d (6.7)	0.83, d (6.7)	0.83, d (6.5)	0.98, d (6.7)
18	0.91, d (7.0)	0.94, d (6.5)	1.09, s	0.92, d (6.5)
18	0.92, d (7.0)	0.97, d (6.5)	0.83, d (6.5)	0.93, d (6.5)

^a^ in CDCl_3_, ^b^ in DMSO-*d*_6_.

## Supplementary Materials

The following are available online at http://www.mdpi.com/2218-273X/8/1/8/s1, Figure S1: HPLC-HRESIMS data of elsinopirin A (**1**), Figure S2: ^1^H NMR spectrum (700 MHz, CDCl_3_) of elsinopirin A (**1**), Figure S3: ^13^C NMR spectrum (175 MHz, CDCl_3_) of elsinopirin A (**1**), Figure S4: COSY NMR spectrum (700 MHz, CDCl_3_) of elsinopirin A (**1**), Figure S5: HSQC NMR spectrum (700 MHz, CDCl_3_) of elsinopirin A (**1**), Figure S6: HMBC NMR spectrum (700 MHz, CDCl_3_) of elsinopirin A (**1**), Figure S7: ROESY NMR spectrum (700 MHz, CDCl_3_) of elsinopirin A (**1**), Figure S8: *J*-resolved NMR spectrum (700 MHz, CDCl_3_) of elsinopirin A (**1**), Figure S9: HPLC-HRESIMS data of elsinopirin B (**2**), Figure S10: ^1^H NMR spectrum (700 MHz, DMSO-*d*_6_) of elsinopirin B (**2**), Figure S11: ^13^C NMR spectrum (175 MHz, DMSO-*d*_6_) of elsinopirin B (**2**), Figure S12: ROESY NMR spectrum (700 MHz, CDCl_3_) of elsinopirin B (**2**), Figure S13: *J*-resolved NMR spectrum (700 MHz, CDCl_3_) of elsinopirin B (**2**), Figure S14: HPLC-HRESIMS data of elsinopirin C (**3**), Figure S15: ^1^H NMR spectrum (500 MHz, DMSO-*d*_6_) of elsinopirin C (**3**), Figure S16: ^13^C NMR spectrum (125 MHz, DMSO-*d*_6_) of elsinopirin C (**3**), Figure S17: ROESY NMR spectrum (500 MHz, CDCl_3_) of elsinopirin C (**3**), Figure S18: HPLC-HRESIMS data of elsinopirin D (**4**), Figure S19: ^1^H NMR spectrum (700 MHz, CDCl_3_) of elsinopirin D (**4**), Figure S20: ^13^C NMR spectrum (175 MHz, CDCl_3_) of elsinopirin D (**4**), Figure S21: HPLC-ESIMS data of elsinopirin A-methyl ester (**6**), Figure S22: ^1^H NMR spectrum (700 MHz, DMSO-*d*_6_) of elsinopirin A-methyl ester (**6**), Figure S23: HPLC-ESIMS data of elsinopirin B-methyl ester (**7**), Figure S24: ^1^H NMR spectrum (700 MHz, DMSO-*d*_6_) of elsinopirin B-methyl ester (**7**), Figure S25: HPLC-ESIMS data of elsinopirin C-methyl ester (**8**), Figure S26: ^1^H NMR spectrum (700 MHz, DMSO-*d*_6_) of elsinopirin C-methyl ester (**8**), Figure S27: HPLC-ESIMS data of elsinopirin D-methyl ester (**9**), Figure S28: ^1^H NMR spectrum (700 MHz, DMSO-*d*_6_) of elsinopirin D-methyl ester (**9**).

## References

[1] Glazowska S.E., Schiller M., Lund O.S., Johnston P.R., Korsgaard M. (2013). First report of elsinoe leaf and fruit spot and *Elsinoe pyri* on apple in Denmark. J. Plant Pathol..

[2] Scheper R.W., Wood P.N., Fisher B.M. (2013). Isolation, spore production and Koch’s postulates of *Elsinoe pyri*. N. Z. Plant Prot..

[3] Fan X.L., Barreto R.W., Groenewald J.Z., Bezerra J.D.P., Pereira O.L., Cheewangkoon R., Mostert L., Tian C.M., Crous P.W. (2017). Phylogeny and taxonomy of the scab and spot anthracnose fungus *Elsinoё* (*Myriangiales, Dothideomycetes*). Stud. Mycol..

[4] Lousberg R.C., Salemink C.A., Weiss U., Batterham T.J. (1969). Pigments of *Elsinoe* species. Part II. Structure of elsinochromes A, B, and C. J. Chem. Soc. C.

[5] Meille S.V., Malpezzi L., Allegra G., Nasini G., Weiss U. (1989). Structure of elsinochrome A: A perylenequinone metabolite. Acta Crystallogr. Sect. C.

[6] Halecker S., Surup F., Kuhnert E., Mohr K.I., Brock N.L., Dickschat J.S., Junker C., Schulz B., Stadler M. (2014). Hymenosetin, a 3-decalinoyltetramic acid antibiotic from cultures of the ash dieback pathogen, *Hymnoscyphus pseudoalbidus*. Phytochemistry.

[7] Surup F., Medjedovic A., Szczygielski M., Schroers H.-J., Stadler M. (2014). Production of trichothecenes by the apple sooty blotch fungus *Microcyclospora tardicrescens*. J. Agric. Food Chem..

[8] Surup F., Medjedovic A., Schroers H.-J., Stadler M. (2015). Production of obionin A and derivatives by the sooty blotch fungus *Microcyclospora malicola*. Planta Med..

[9] Ondeyka J.G., Giacobbe R.A., Bills G.F., Cuadrillero C., Schmatz D., Goetz M.A., Zink D.L., Singh S.B. (1998). Coprophillin: An anticoccidial agent produced by a dung inhabiting fungus. Bioorg. Med. Chem. Lett..

[10] Surup F., Kuhnert E., Böhm A., Pendzialek T., Solga D., Wiebach V., Engler H., Berkessel A., Stadler M., Kalesse M. (2017). The rickiols, 20-, 22-, and 24-membered macrolides from the ascomycete *Hypoxylon rickii*. Chem. Eur. J..

[11] Li G., Kusari S., Spiteller M. (2014). Natural products containing ‘decalin’ motif in microorganisms. Nat. Prod. Rep..

[12] Kuramoto M., Yamada K., Shikano M., Yazawa K., Arimoto H., Okamura T., Uemura D. (1997). Tanzawaic acids A, B, C, and D: Inhibitors of superoxide anion production from *Penicillium citrinum*. Chem. Lett..

[13] Sandjo L.P., Thines E., Opatz T., Schüffler A. (2014). Tanzawaic acids I–L: Four new polyketides from *Penicillium* sp.. Beilstein J. Org. Chem..

[14] Tabata N., Tomoda H., Iwai Y., Omura S. (1993). Hynapenes A, B, and C, new anticoccidial agents produced by *Penicillium* sp.. J. Antibiot..

