# Supplementary material for: Elsinopirins A–D, Decalin Polyketides from the Ascomycete Elsinoё pyri
Source: Biomolecules. 2018 Feb 5;8(1):8. doi: 10.3390/biom8010008 (PMC5871977; doi:10.3390/biom8010008)
Supplement: Supplementary file 1 [file biomolecules-08-00008-s001.pdf]

Article

Elsinopirins A-D, decalin polyketides from the ascomycete *Elsinoë piri*

Frank Surup[1,2], Kathrin Pommerehne[1,4], Hans-Josef Schroers[3] and Marc Stadler[1, 2,*]

[1] Microbial Drugs, Helmholtz Centre for Infection Research GmbH (HZI), Inhoffenstraße 7, 38124 Braunschweig, Germany; frank.surup@helmholtz-hzi.de (F.S.); k.pommerehne@tu-braunschweig.de (K.P.)

[2] German Centre for Infection Research Association (DZIF), partner site Hannover-Braunschweig, Inhoffenstraße 7, 38124 Braunschweig, Germany

[3] Agricultural Institute of Slovenia, Hacquetova ulica 17, 1000 Ljubljana, Slovenia; hans.schroers@kis.si

[4] Institute of Biochemical Engineering, Technical University Braunschweig, Rebenring 56, 38106 Braunschweig; Germany; k.pommerehne@tu-braunschweig.de

* Correspondence: marc.stadler@helmholtz-hzi.de; Tel.: +49-531-6181-4240

--- Supporting Information---

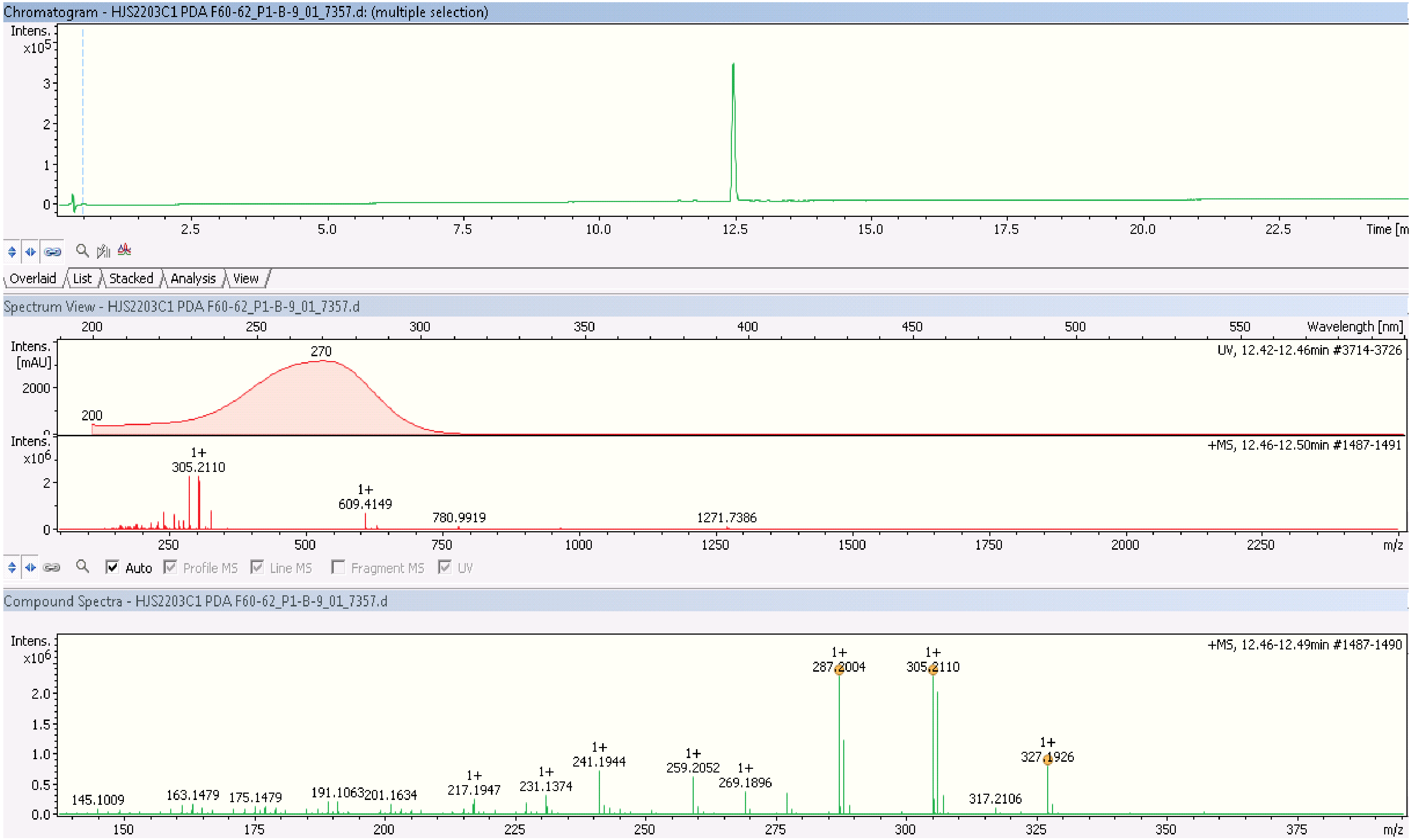

Figure S1: HPLC-HRESIMS data of elsinopirin A (**1**).

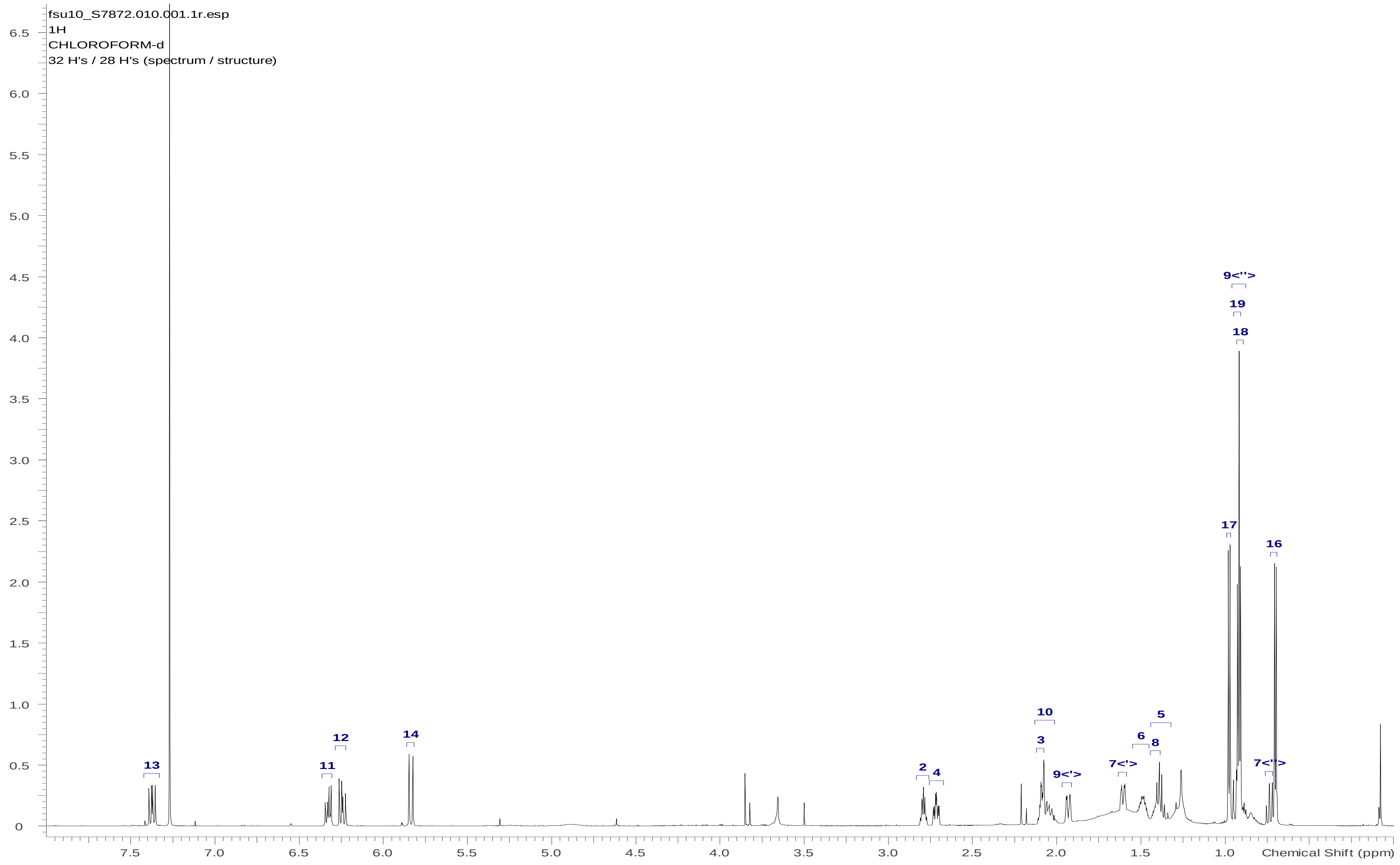

Figure S2: ¹H NMR spectrum (700 MHz, $CDCl_3$) of elsinopirin A (**1**).

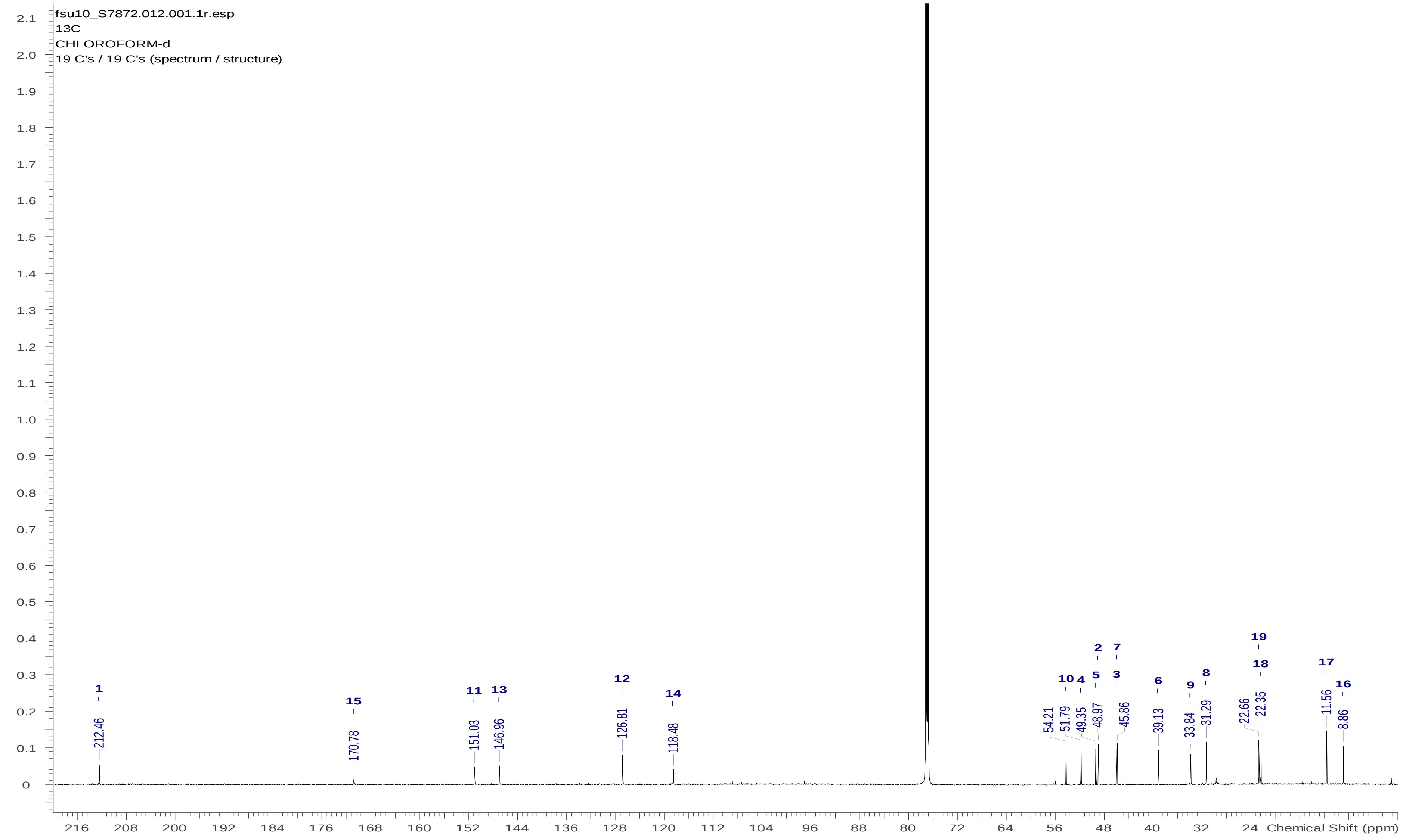

Figure S3: ^{13}C NMR spectrum (175 MHz, $CDCl_3$) of elsinopirin A (**1**).

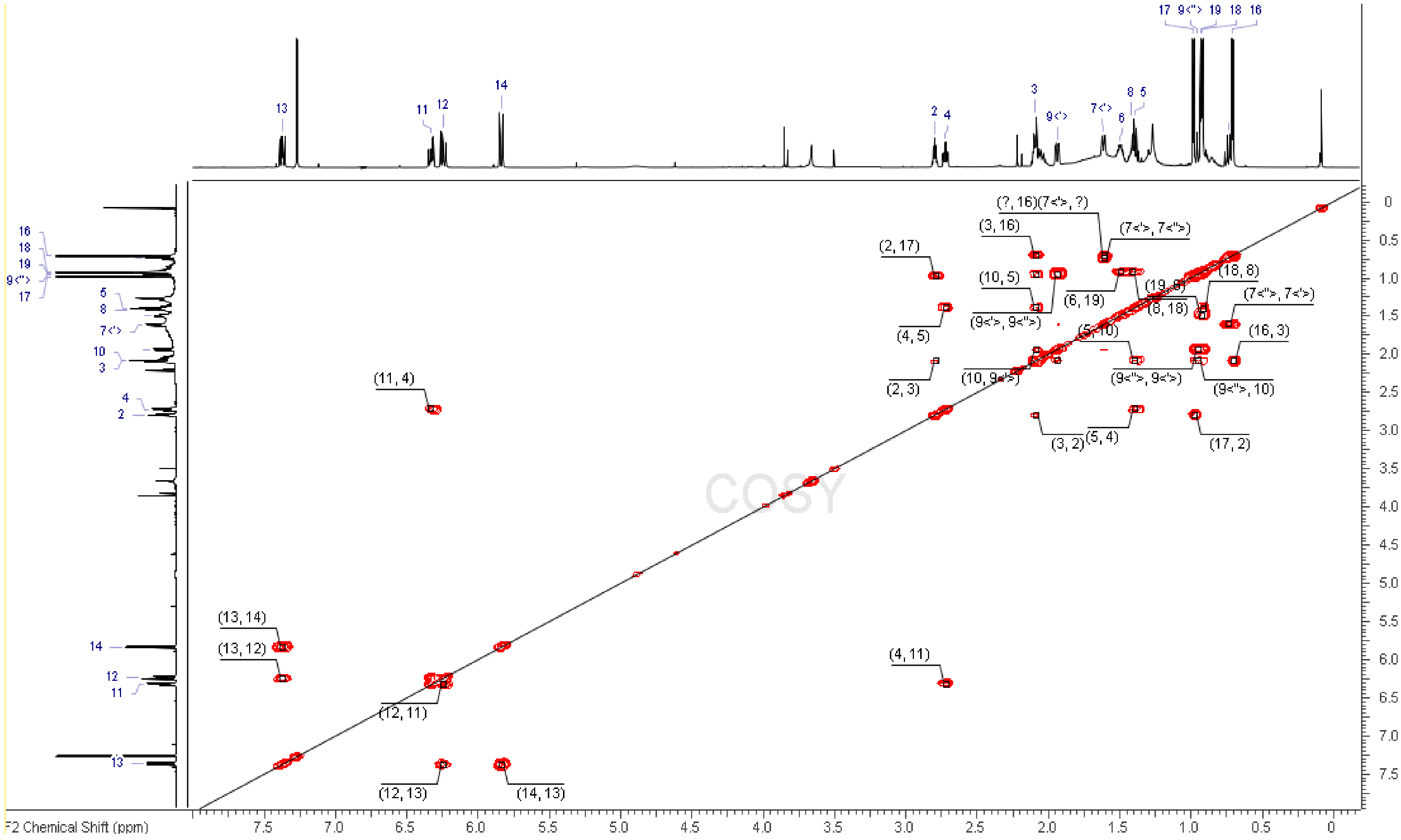

Figure S4: COSY NMR spectrum (700 MHz, $CDCl_3$) of elsinopirin A (**1**).

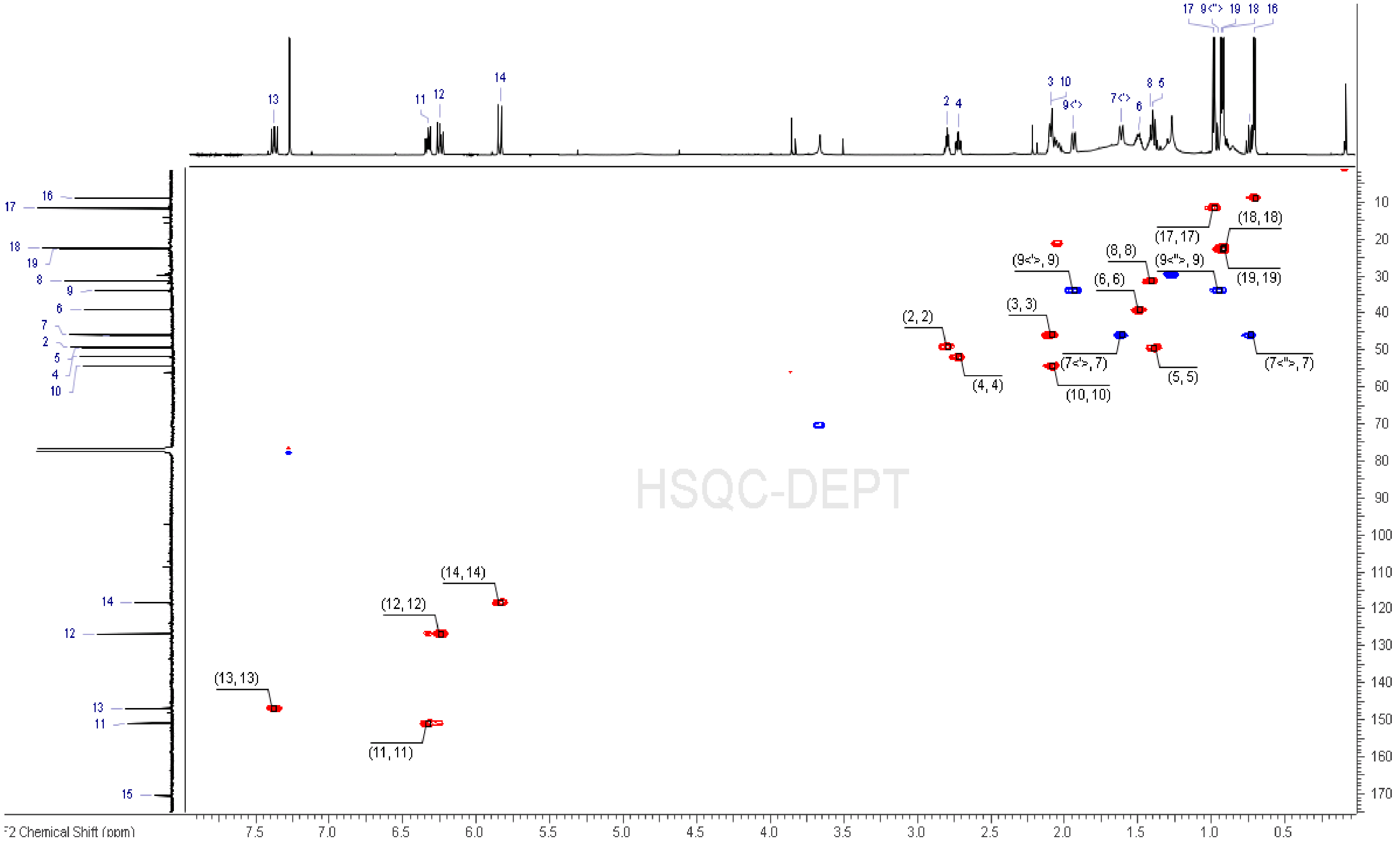

Figure S5: HSQC NMR spectrum (700 MHz, $CDCl_3$) of elsinopirin A (**1**).

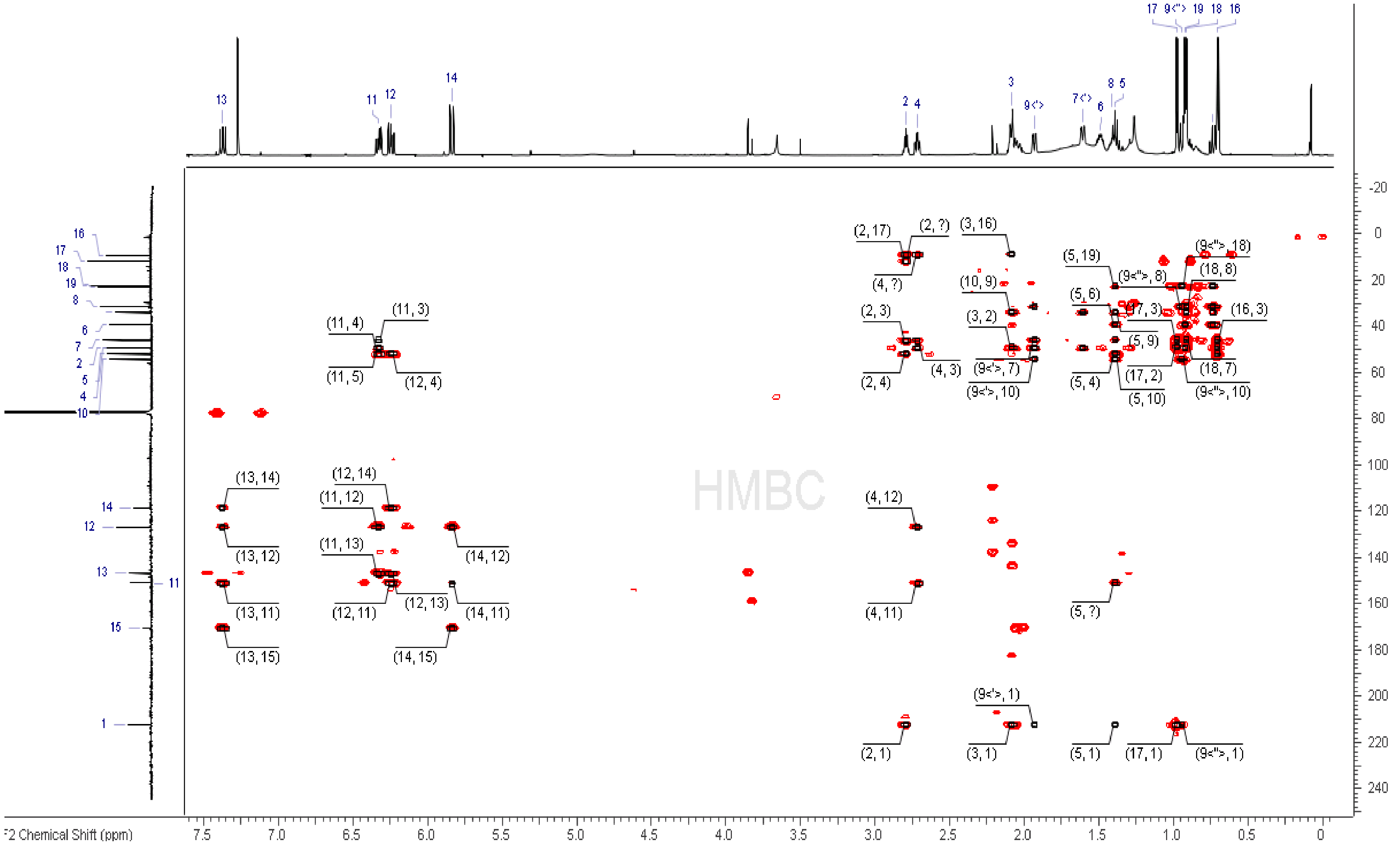

Figure S6: HMBC NMR spectrum (700 MHz, $CDCl_3$) of elsinopirin A (**1**).

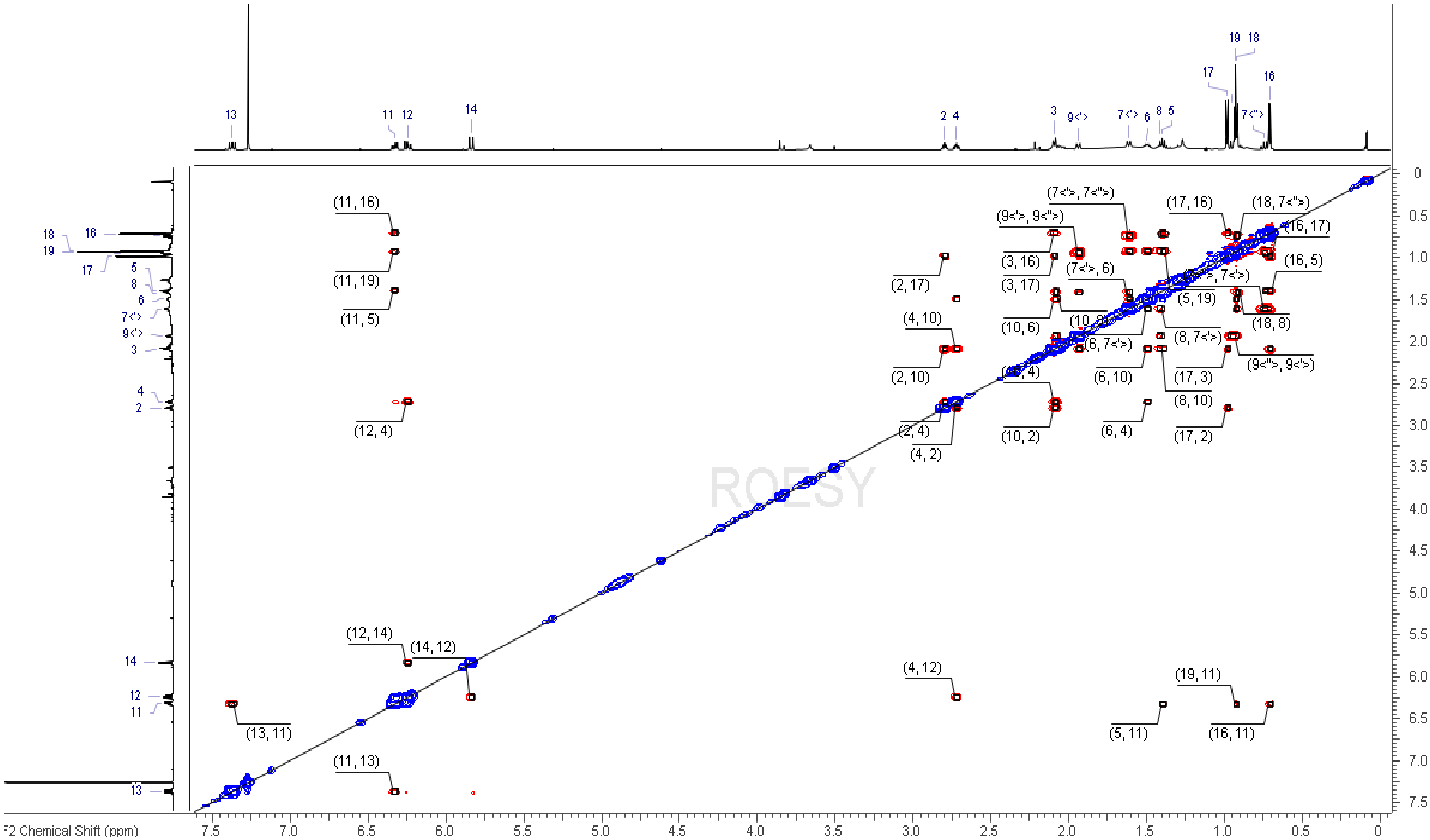

Figure S7: ROESY NMR spectrum (700 MHz, $CDCl_3$) of elsinopirin A (**1**).

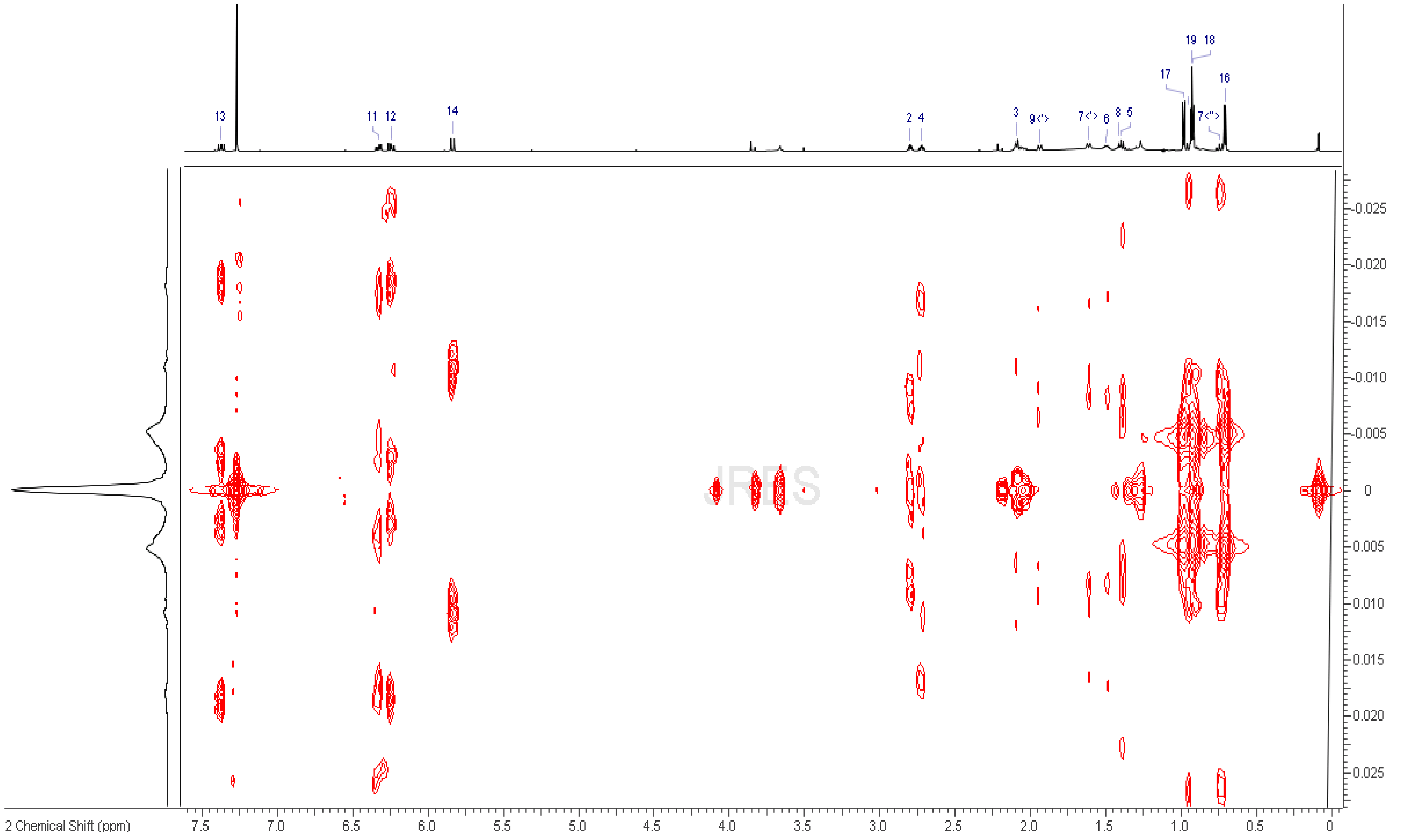

Figure S8: *J*-resolved NMR spectrum (700 MHz, $CDCl_3$) of elsinopirin A (**1**).

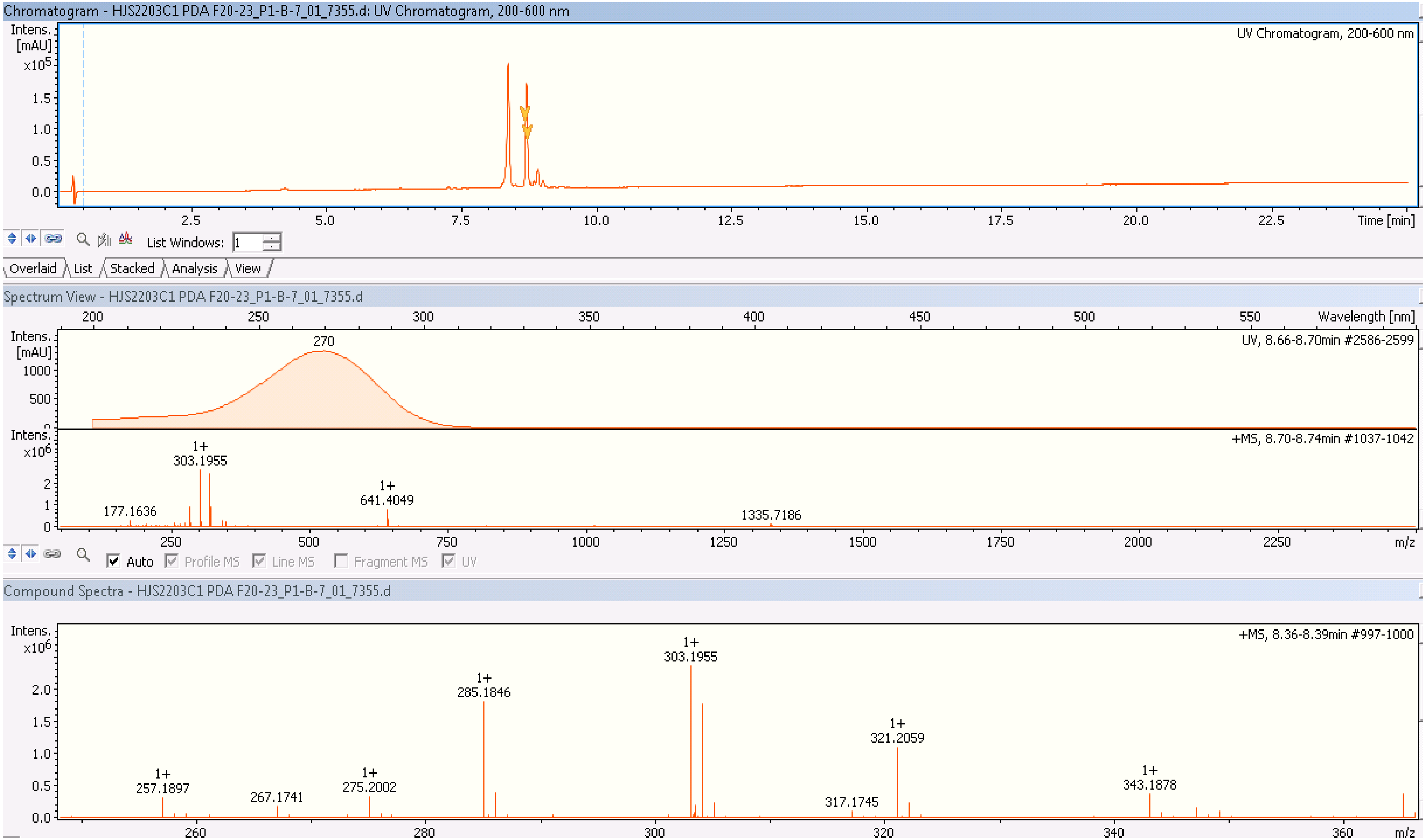

Figure S9: HPLC-HRESIMS data of elsinopirin B (**2**).

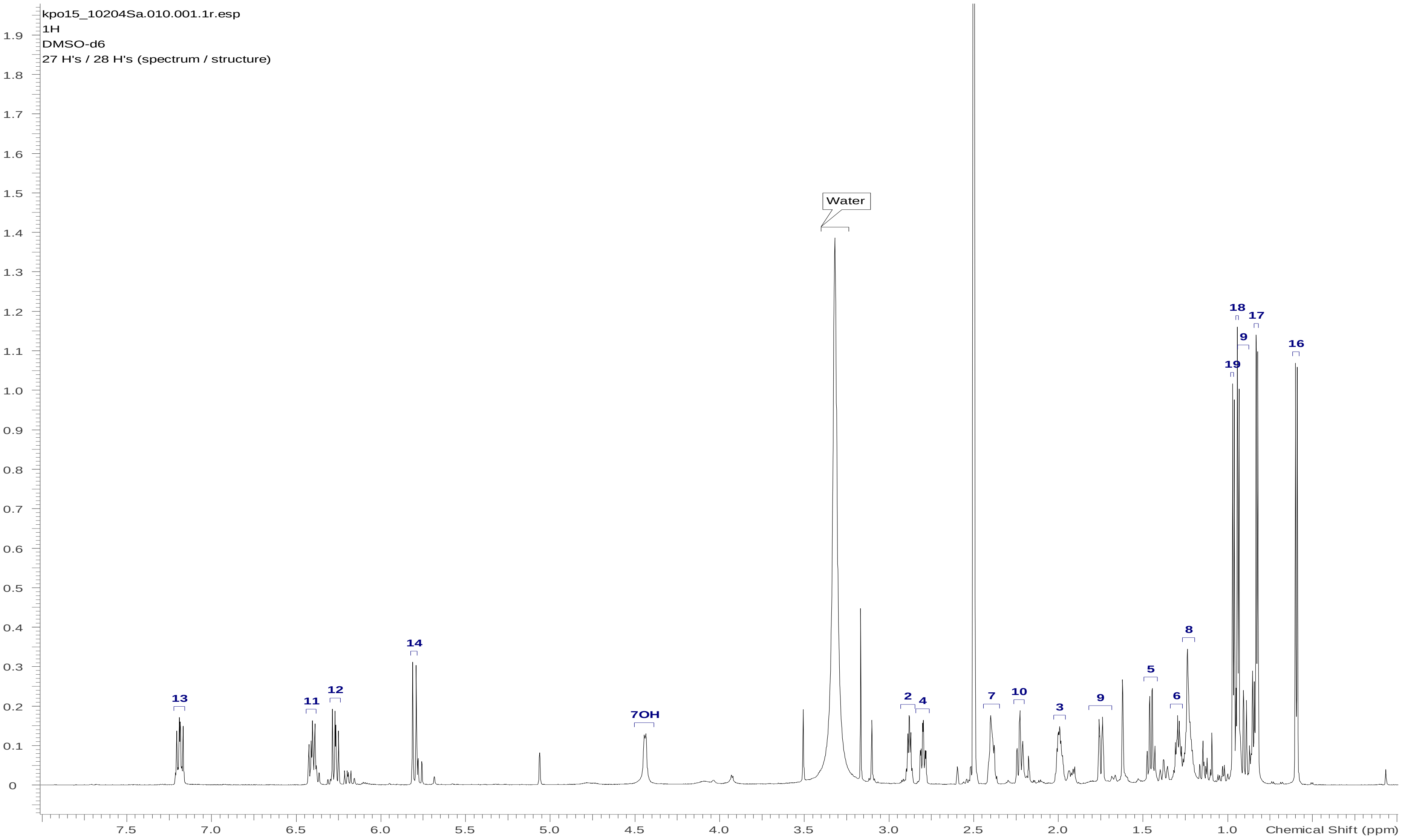

Figure S10: ^{1}H NMR spectrum (700 MHz, DMSO-*d*6) of elsinopirin B (**2**).

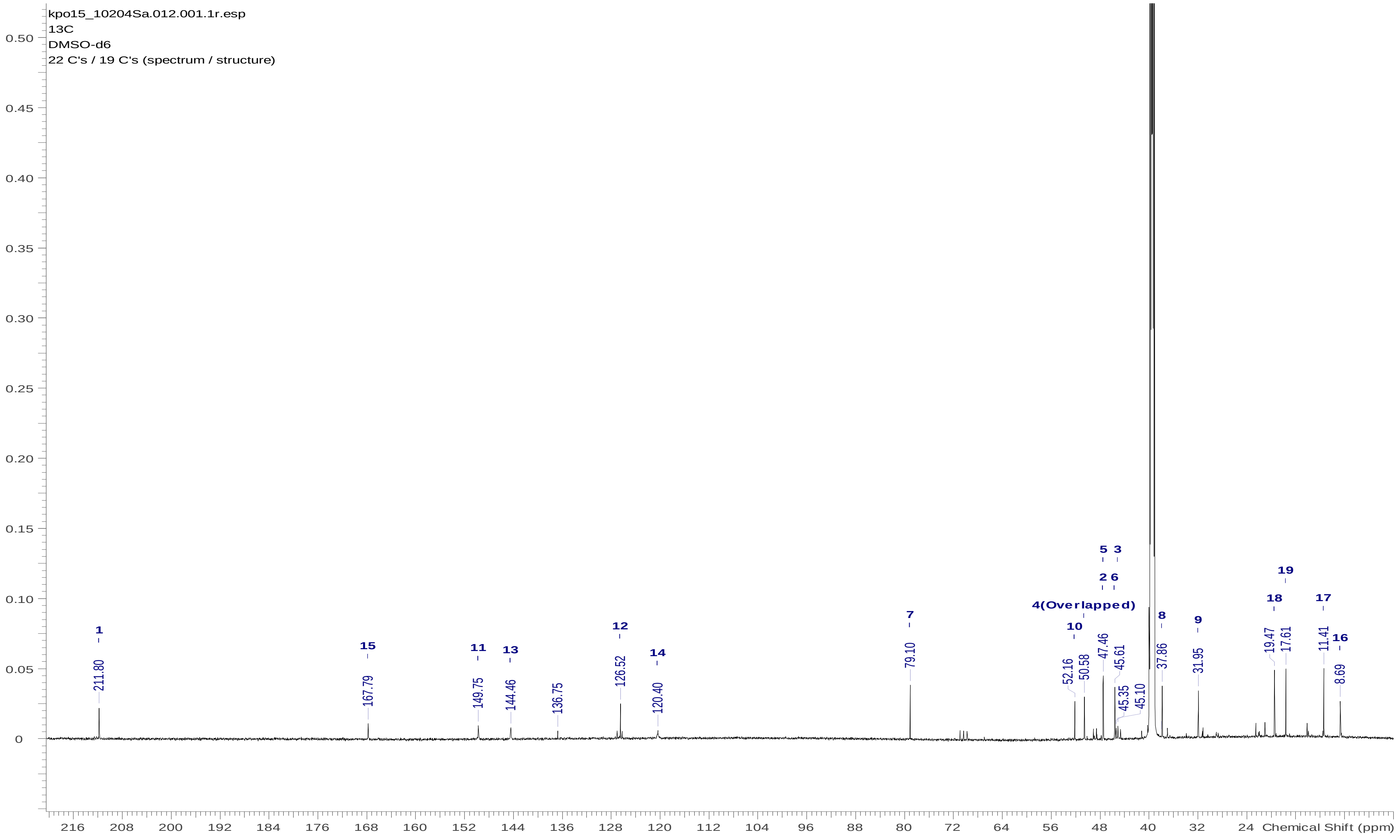

Figure S11: ^{13}C NMR spectrum (175 MHz, DMSO-d_6) of elsinopirin B (**2**).

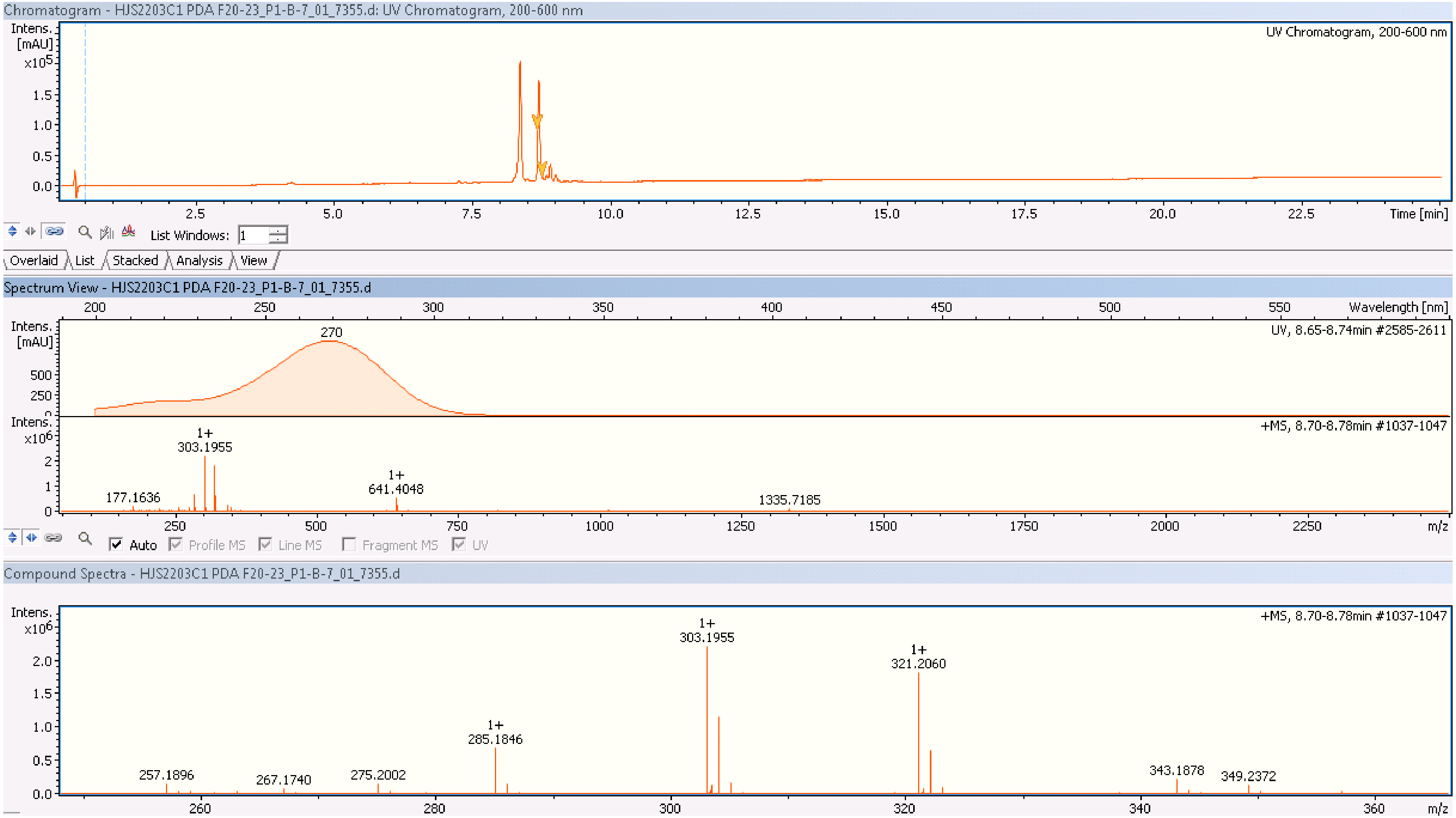

Figure S12: HPLC-HRESIMS data of elsinopirin C (**3**).

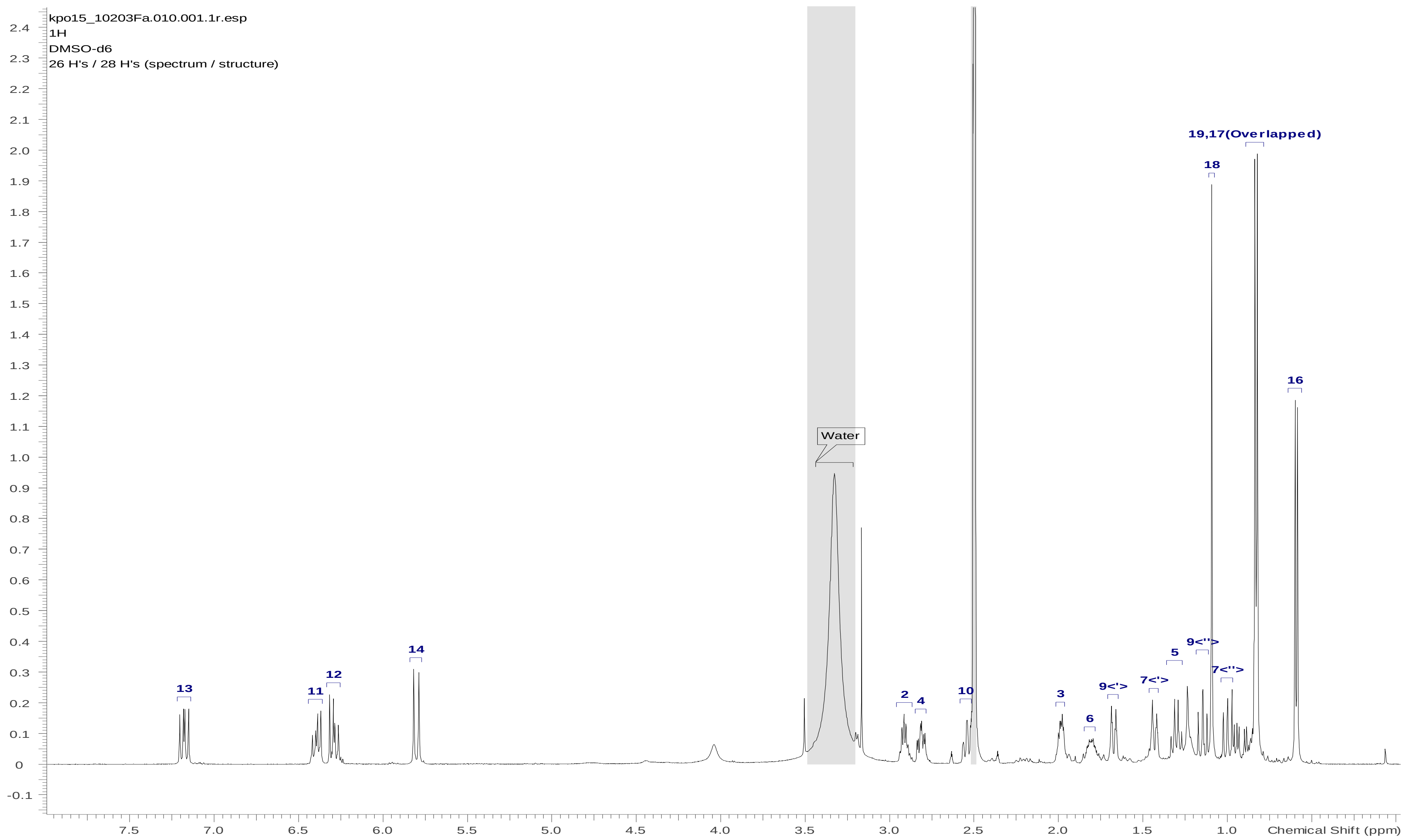

Figure S13: ^{1}H NMR spectrum (500 MHz, DMSO-d_6) of elsinopirin C (**3**).

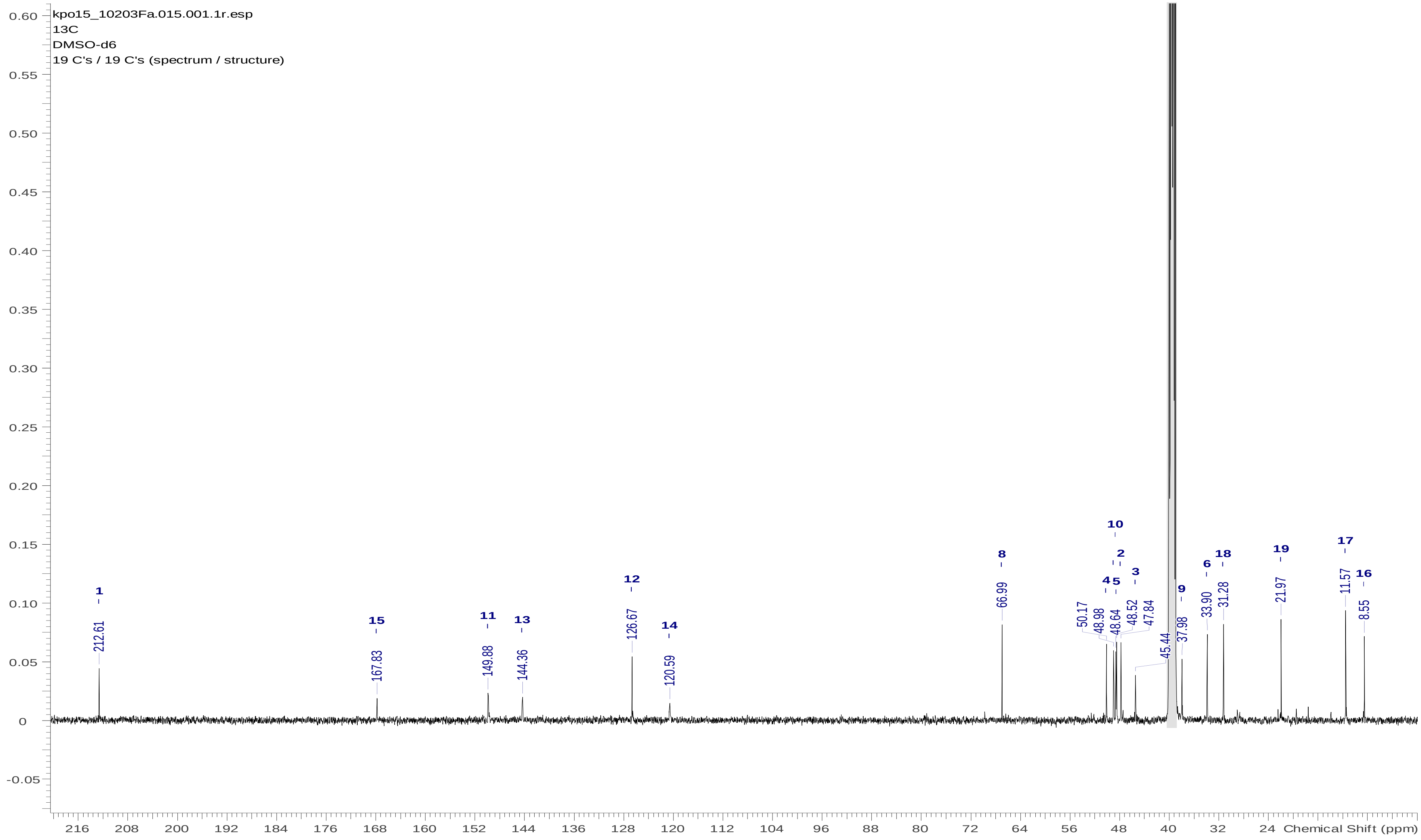

Figure S14: ^{13}C NMR spectrum (125 MHz, DMSO-*d*$_6$) of elsinopirin C (**3**).

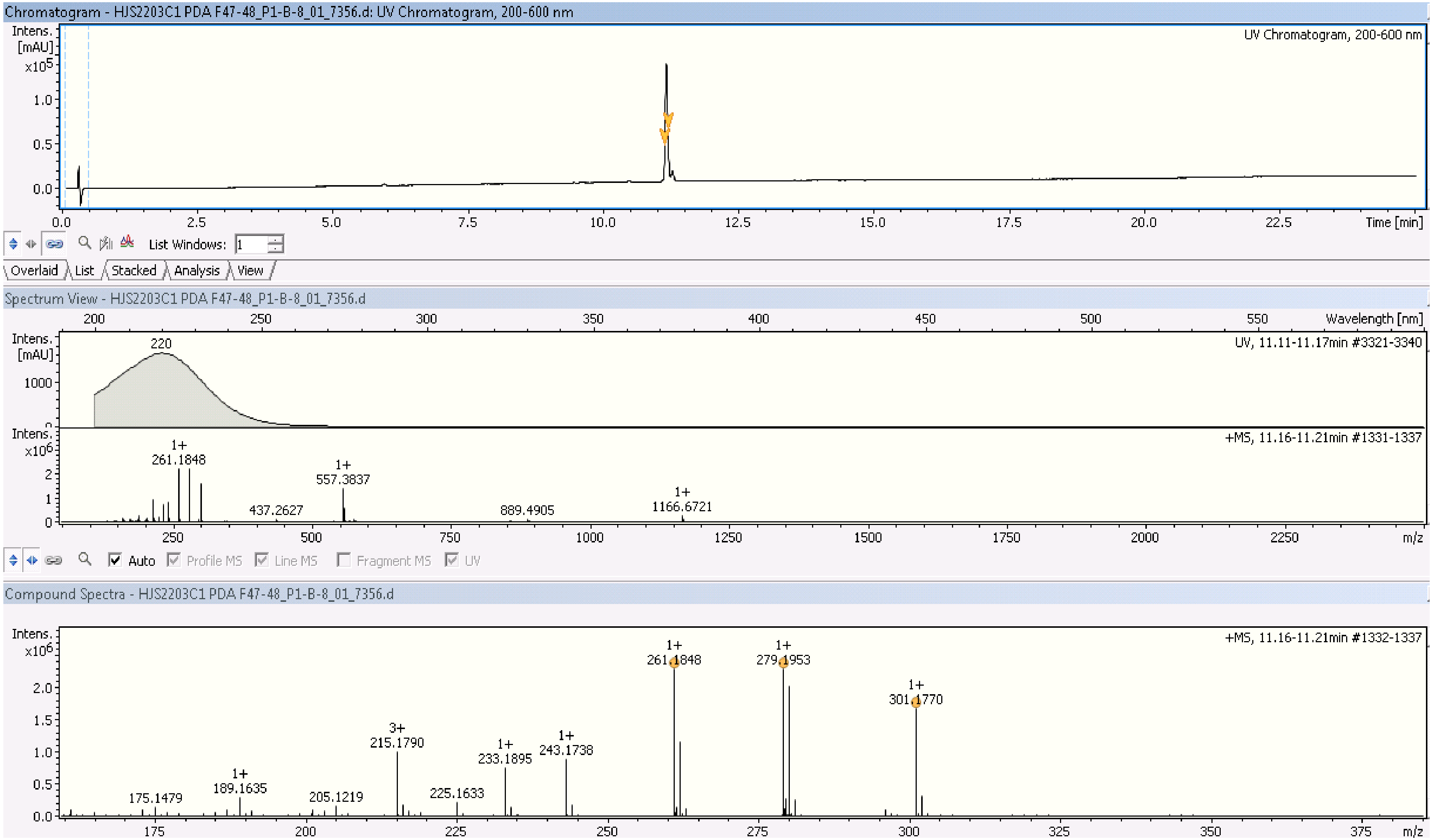

Figure S15: HPLC-HRESIMS data of elsinopirin D (**4**).

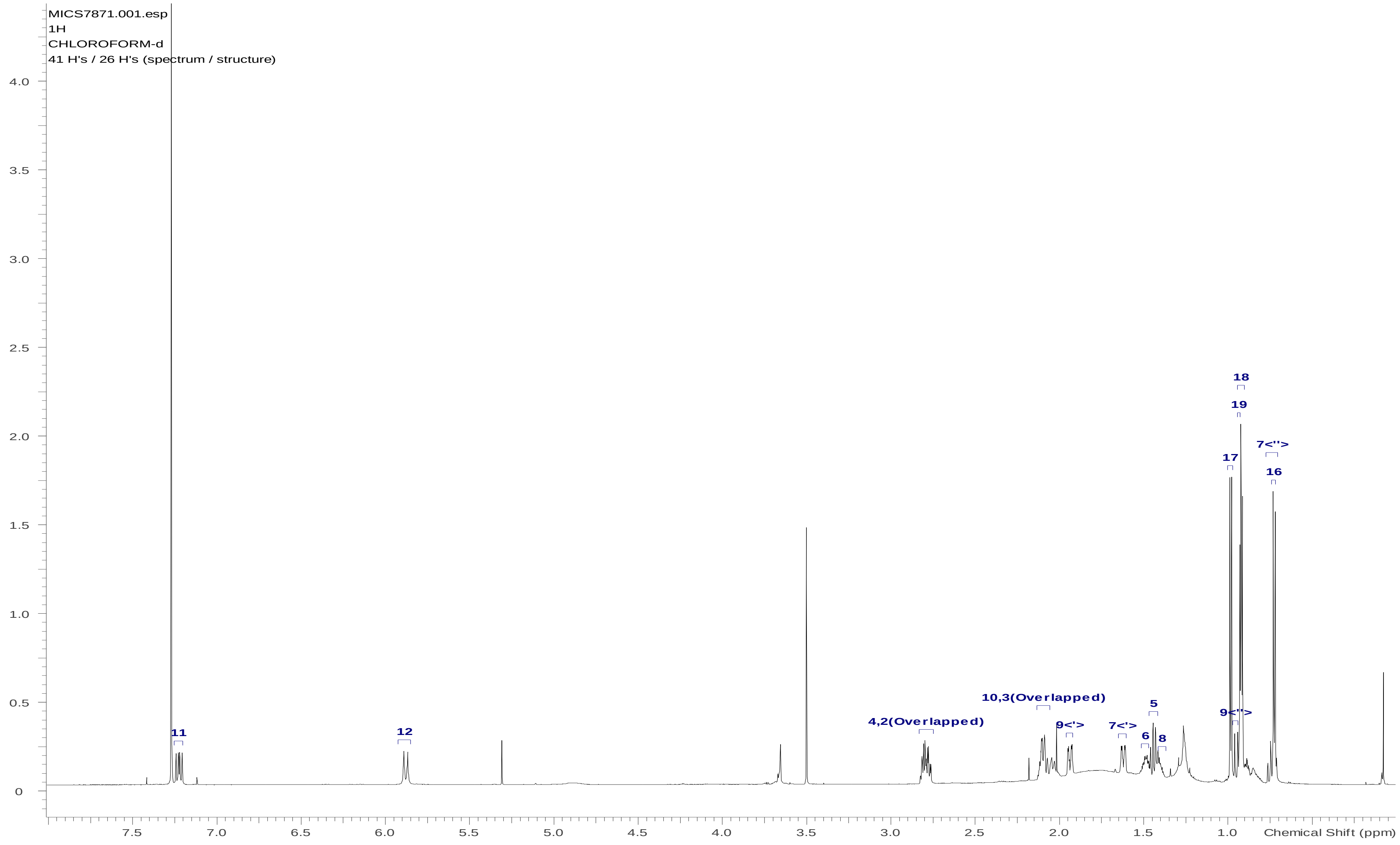

Figure S16: ^1H NMR spectrum (700 MHz, $CDCl_3$) of elsinopirin D (**4**).

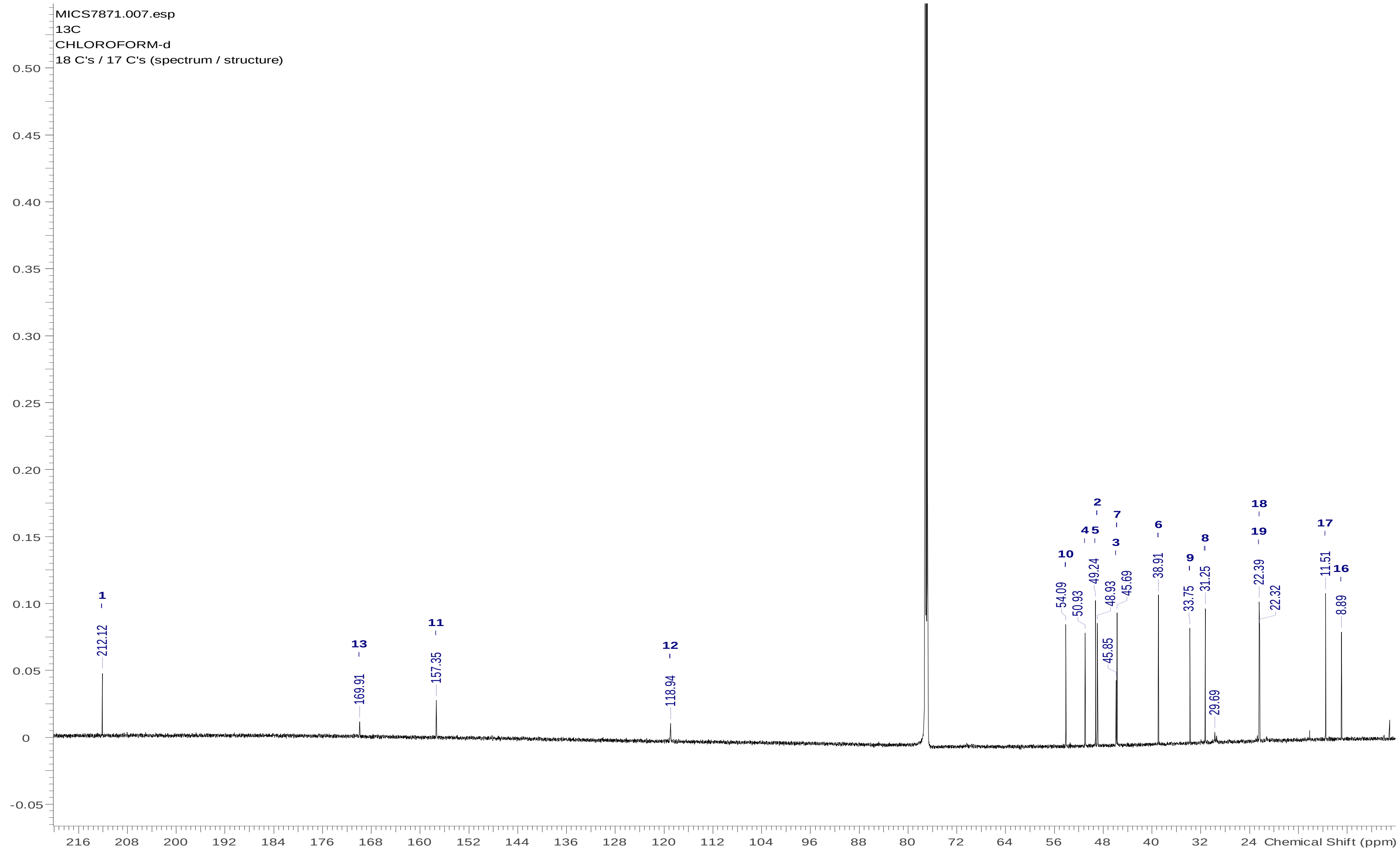

Figure S17: ^{13}C NMR spectrum (175 MHz, $CDCl_3$) of elsinopirin D (**4**).

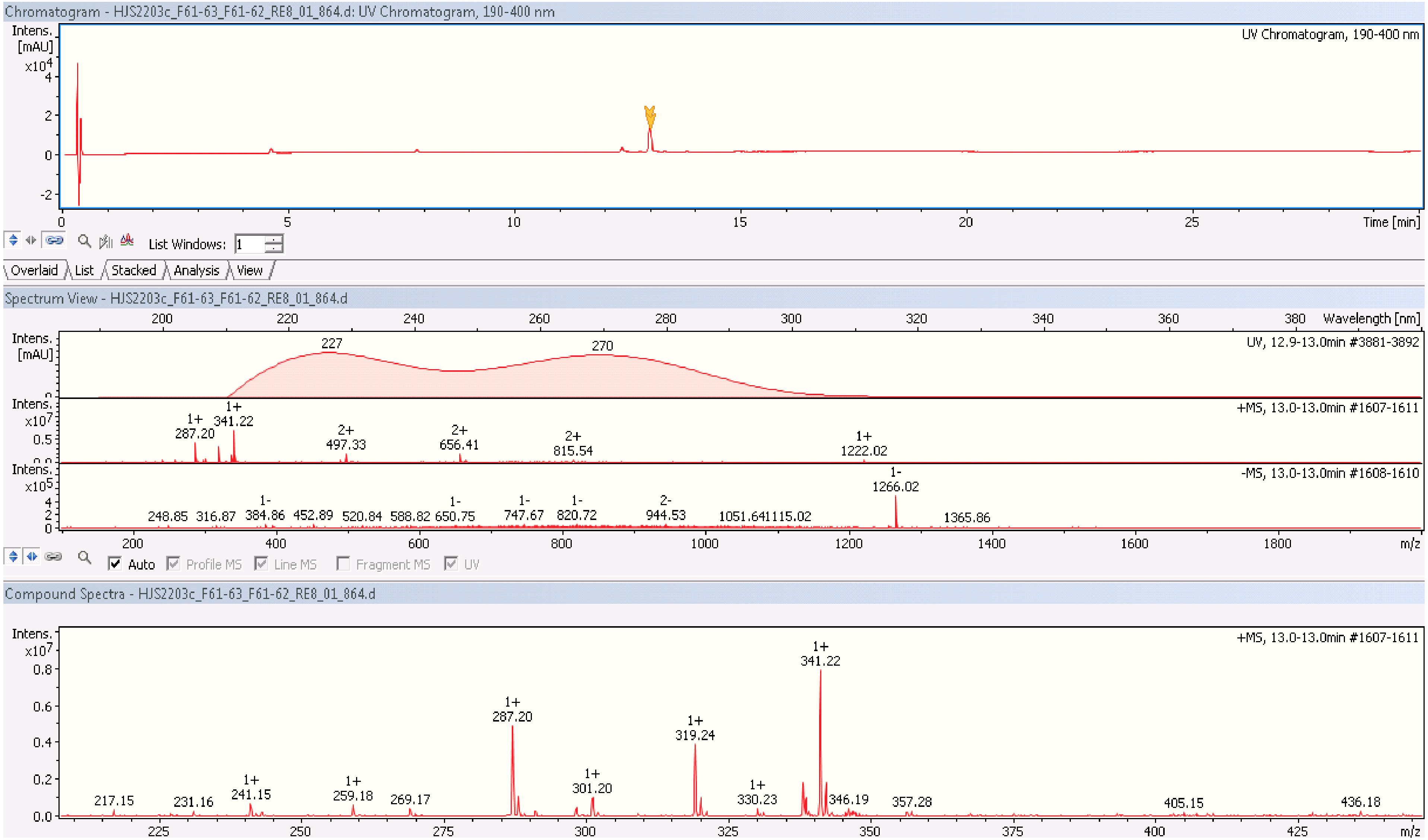

Figure S18: HPLC-ESIMS data of elsinopirin A-methylester (**6**).

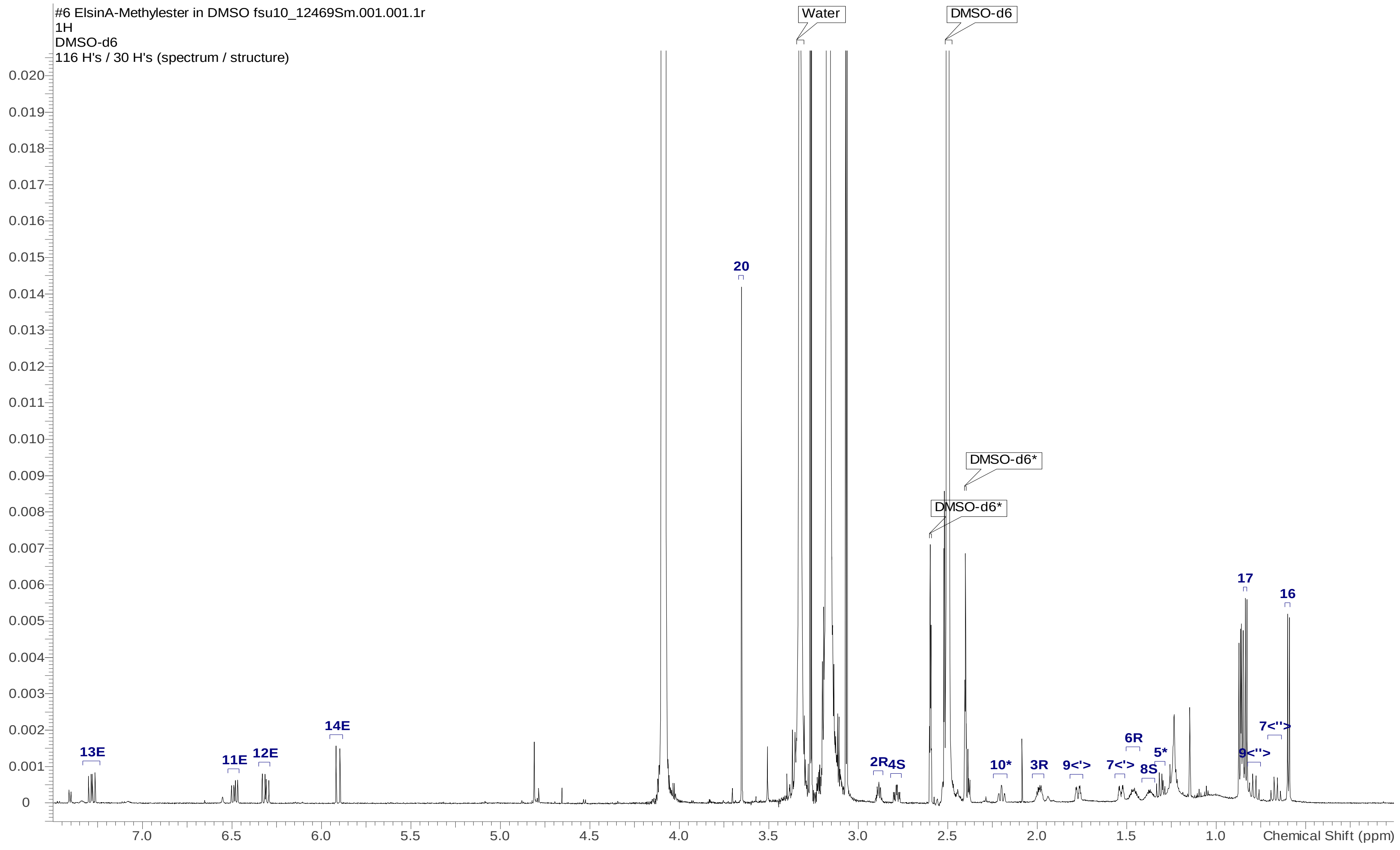

Figure S19: ^{1}H NMR spectrum (700 MHz, DMSO-d_6) of elsinopirin A-methylester (**6**).

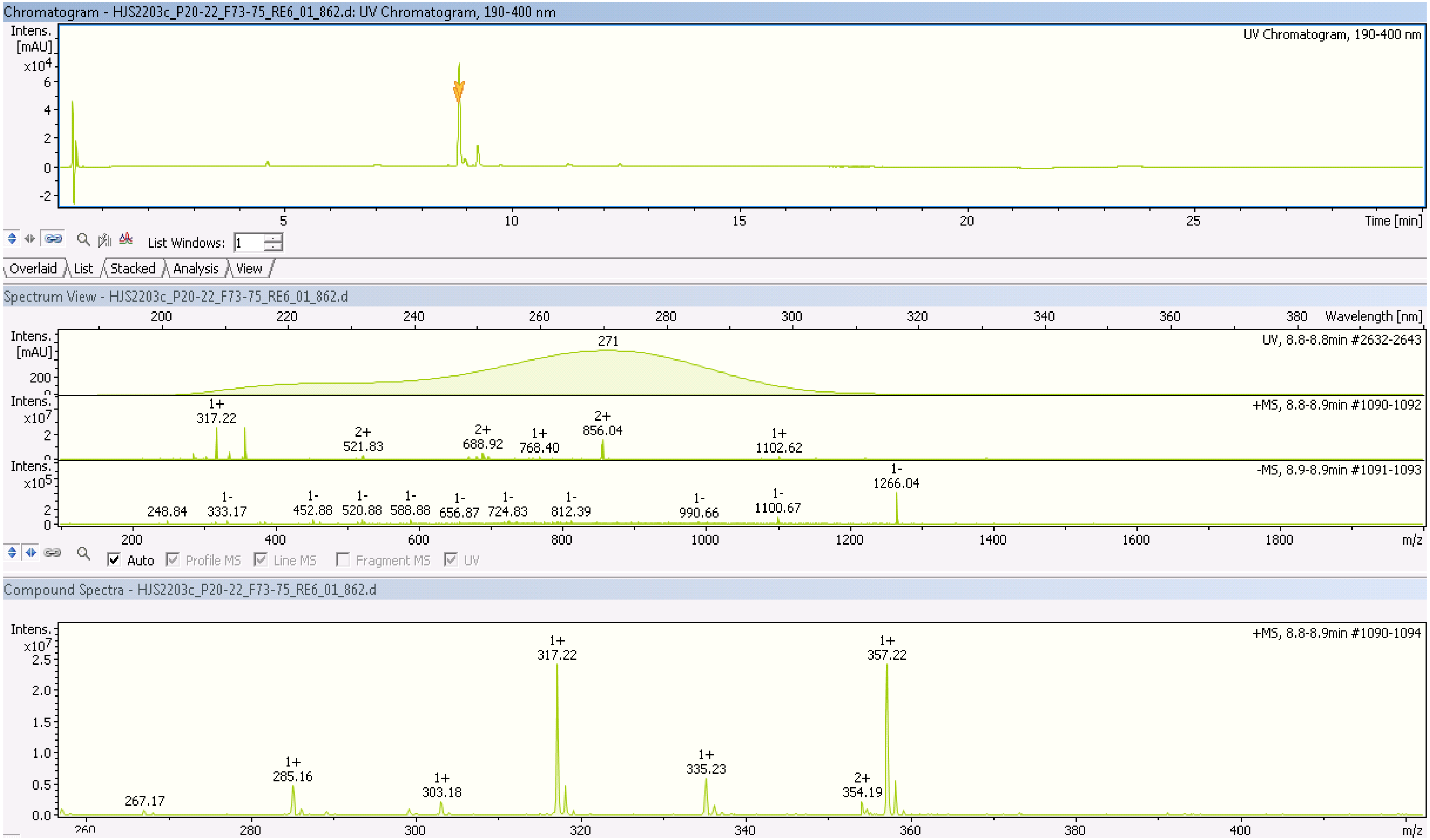

Figure S20: HPLC-ESIMS data of elsinopirin B-methylester (**7**).

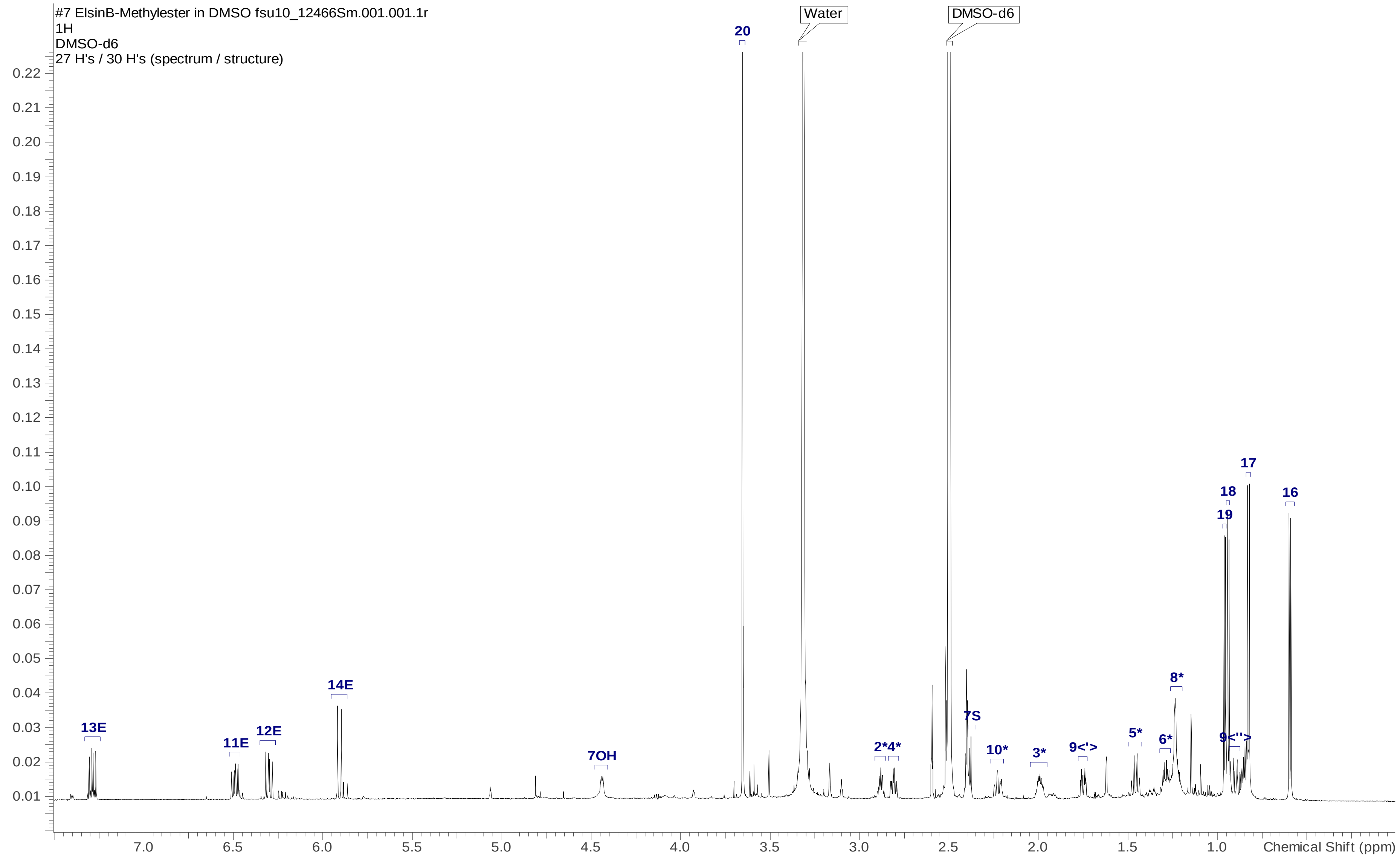

Figure S21: ^{1}H NMR spectrum (700 MHz, DMSO-d_6) of elsinopirin B-methylester (**7**).

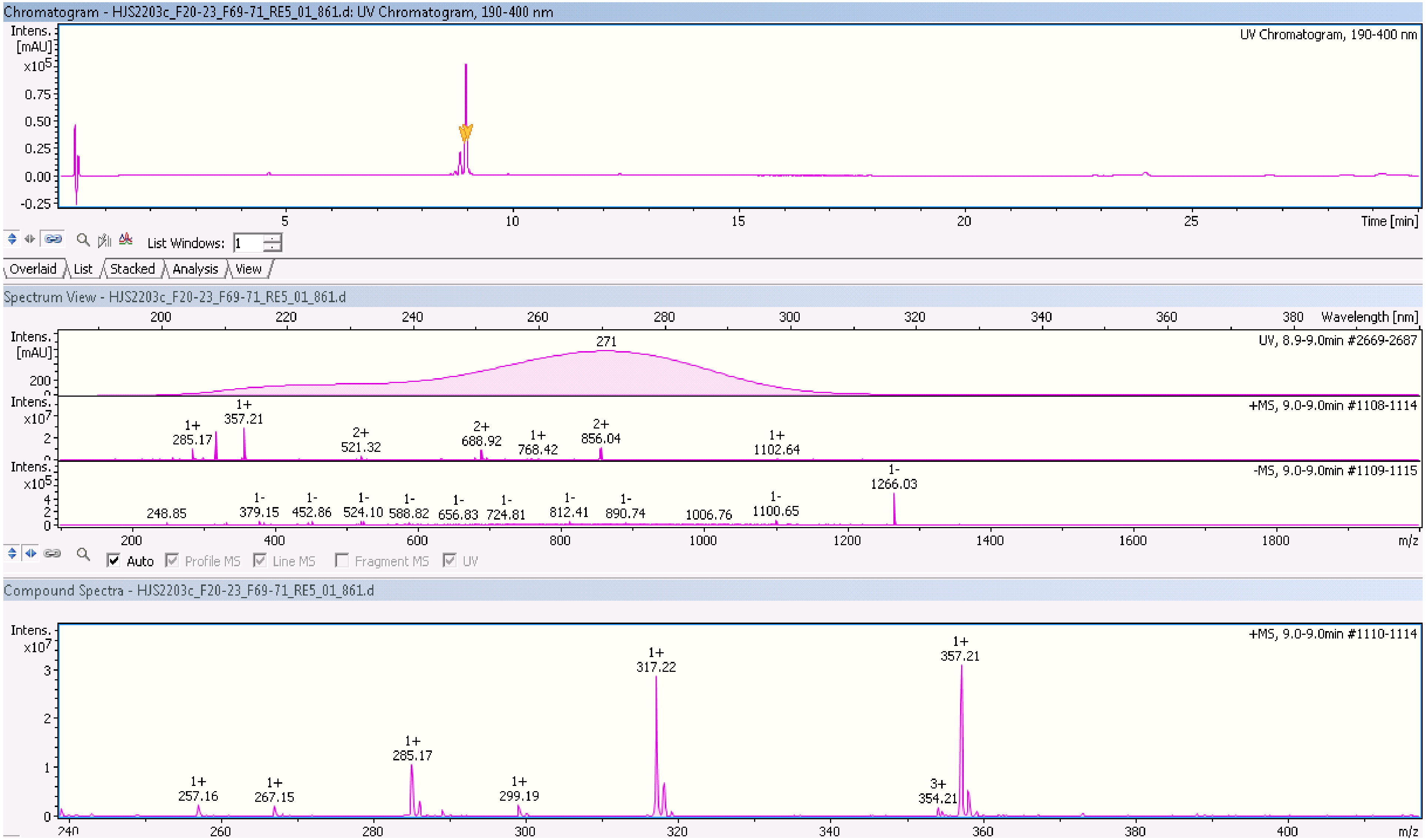

Figure S22: HPLC-ESIMS data of elsinopirin C-methylester (**8**).

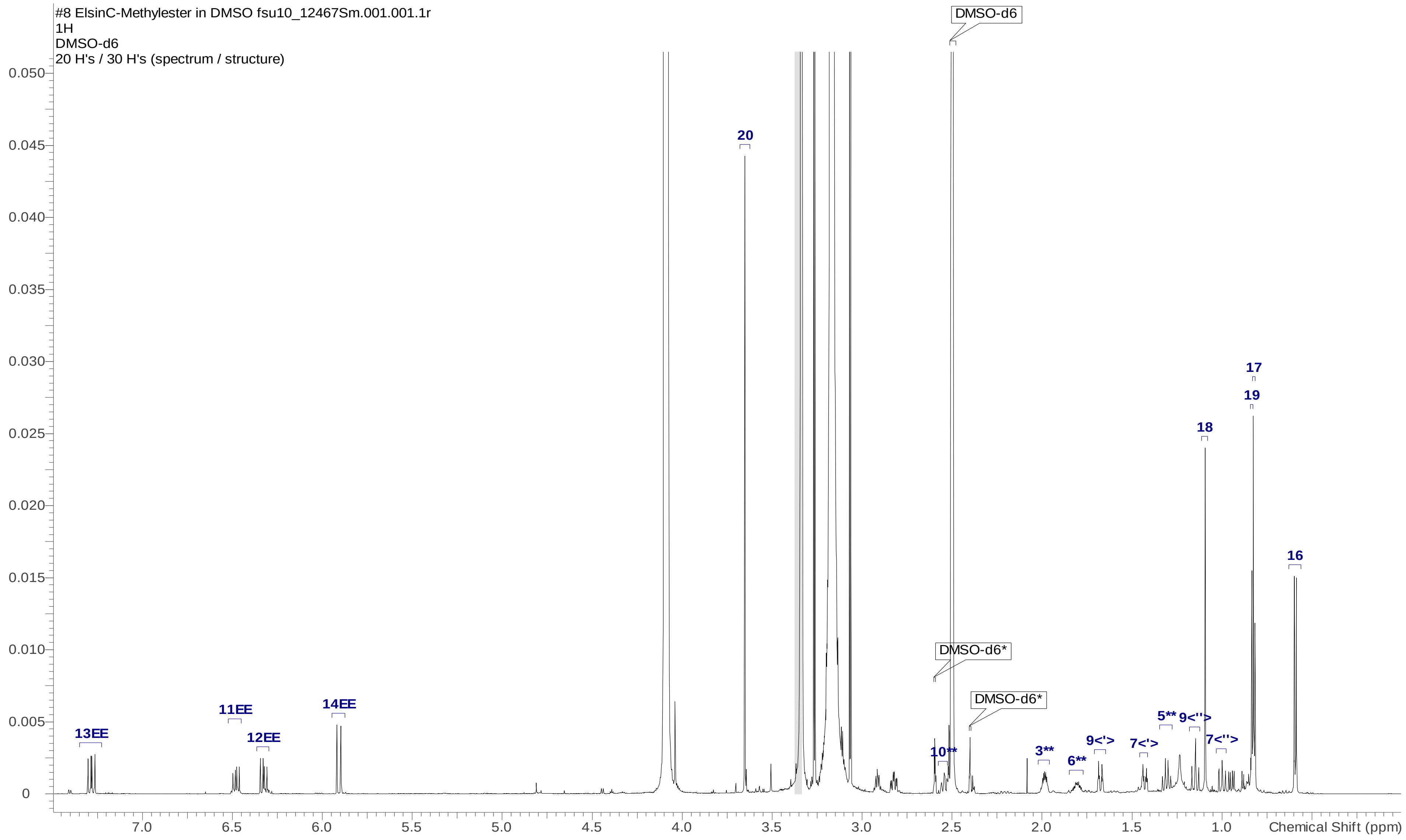

Figure S23: ^{1}H NMR spectrum (700 MHz, DMSO-d_6) of elsinopirin C-methylester (**8**).

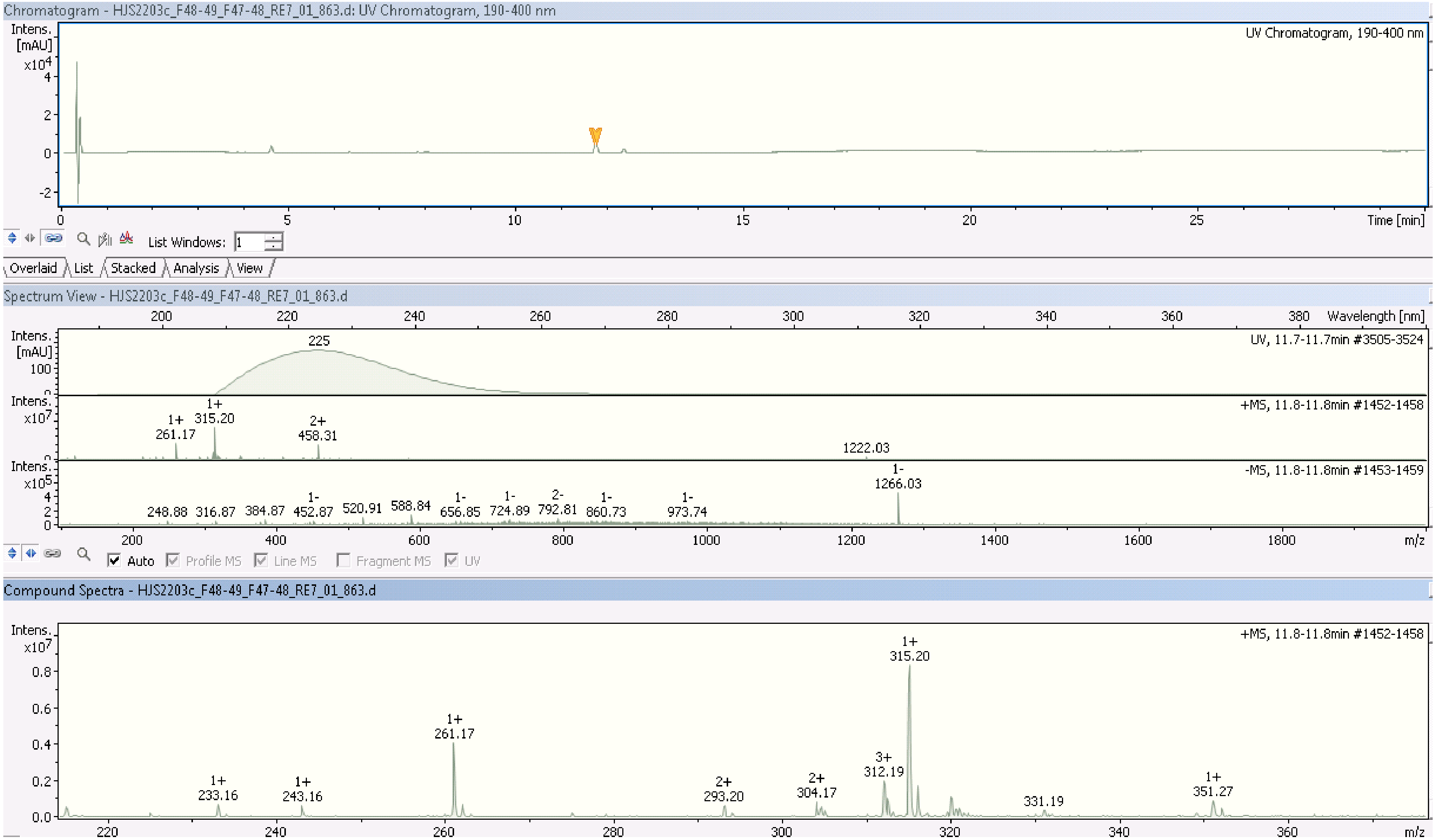

Figure S24: HPLC-ESIMS data of elsinopirin D-methylester (**9**).

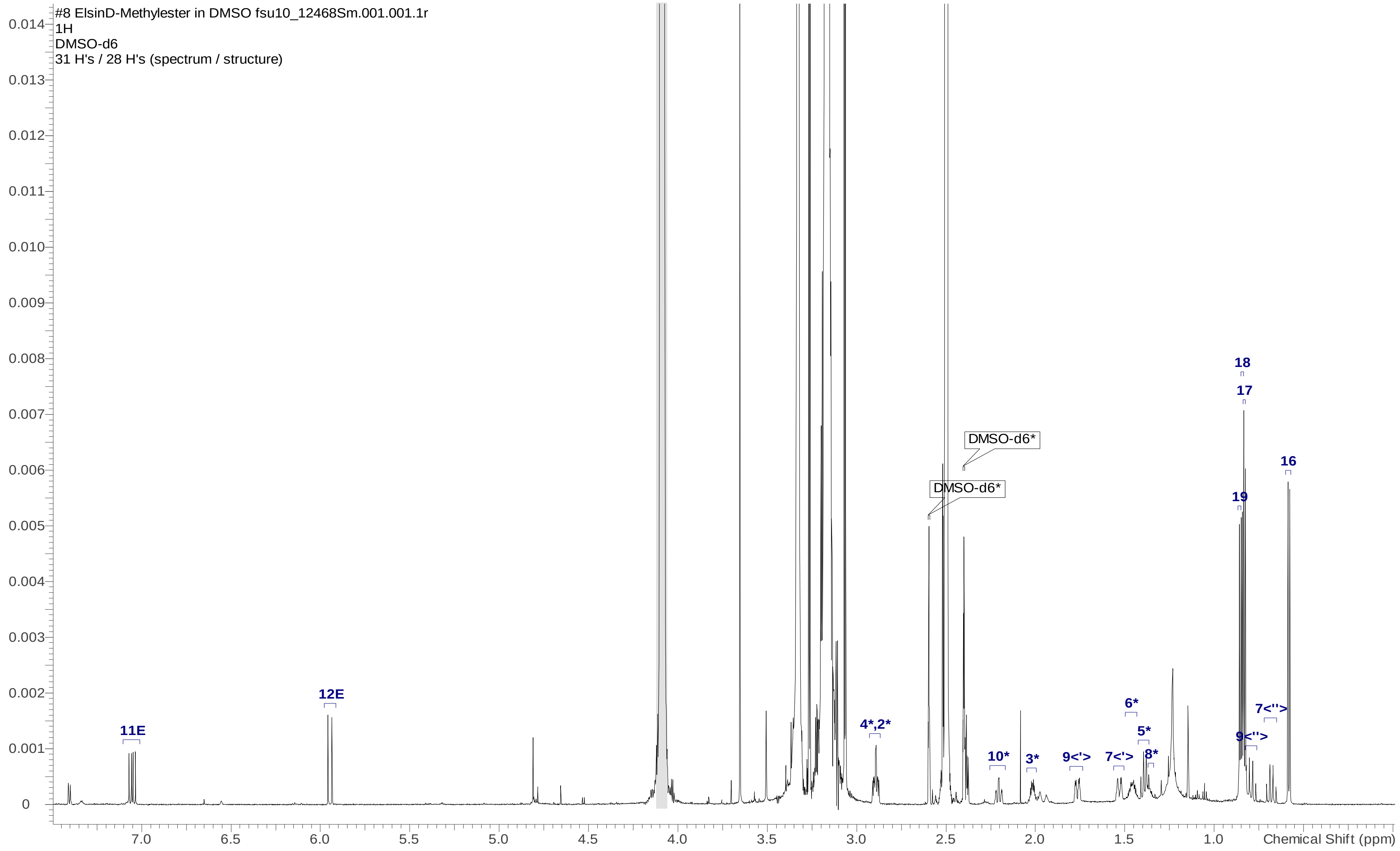

Figure S25: ^{1}H NMR spectrum (700 MHz, DMSO-*d*6) of elsinopirin D-methylester (**9**).
